# SUN1/2 controls macrophage polarization via modulating nuclear size and stiffness

**DOI:** 10.1038/s41467-023-42187-5

**Published:** 2023-10-12

**Authors:** Shi Jiao, Chuanchuan Li, Fenghua Guo, Jinjin Zhang, Hui Zhang, Zhifa Cao, Wenjia Wang, Wenbo Bu, Mobin Lin, Junhong Lü, Zhaocai Zhou

**Affiliations:** 1 State Key Laboratory of Genetic Engineering, School of Life Sciences, Zhongshan Hospital, Fudan University, Shanghai, 200438 China; 2 CAS Center for Excellence in Molecular Cell Science, Institute of Biochemistry and Cell Biology, Shanghai Institutes for Biological Sciences, Chinese Academy of Sciences, University of Chinese Academy of Sciences, Shanghai, 200031 China; 3 Department of General Surgery, Hua’shan Hospital, Fudan University Shanghai Medical College, Shanghai, 200040 China; 4 Shanghai Advanced Research Institute, Chinese Academy of Sciences, Shanghai, 201203 China; 5 Department of Stomatology, Department of Medical Ultrasound, Shanghai Tenth People’s Hospital, Department of Biochemistry and Molecular Biology, Tongji University School of Medicine, Shanghai, 200072 China; 6 Department of Materials Science and State Key Laboratory of Molecular Engineering of Polymers, Fudan University, Shanghai, 200433 China; 7 Department of General Surgery, Yangpu Hospital, Tongji University School of Medicine, Shanghai, 200090 China; 8 College of Pharmacy, Binzhou Medical University, Yantai, 264003 China; 9 Collaborative Innovation Center for Cancer Personalized Medicine, School of Public Health, Nanjing Medical University, Nanjing, 211166 China; 10 Present Address: Human Oncology and Pathogenesis Program, Memorial Sloan Kettering Cancer Center, 417 E 68th St, New York, NY 10065 USA

**Keywords:** Monocytes and macrophages, Nuclear envelope

## Abstract

Alteration of the size and stiffness of the nucleus triggered by environmental cues are thought to be important for eukaryotic cell fate and function. However, it remains unclear how context-dependent nuclear remodeling occurs and reprograms gene expression. Here we identify the nuclear envelope proteins SUN1/2 as mechano-regulators of the nucleus during M1 polarization of the macrophage. Specifically, we show that LPS treatment decreases the protein levels of SUN1/2 in a CK2-βTrCP-dependent manner to shrink and soften the nucleus, therefore altering the chromatin accessibility for M1-associated gene expression. Notably, the transmembrane helix of SUN1/2 is solely required and sufficient for the nuclear mechano-remodeling. Consistently, SUN1/2 depletion in macrophages facilitates their phagocytosis, tissue infiltration, and proinflammatory cytokine production, thereby boosting the antitumor immunity in mice. Thus, our study demonstrates that, in response to inflammatory cues, SUN1/2 proteins act as mechano-regulators to remodel the nucleus and chromatin for M1 polarization of the macrophage.

## Abstract (web summary)

Stiffness and size of the nucleus may affect the function of specific cell types. Here the authors show that LPS treatment of macrophages affects the nucleus stiffness and size involving nuclear envelope proteins SUN1/2, chromatin accessibility and M1 associated gene expression.

## Introduction

As a defining feature of eukaryotic cells, the nucleus deploys a physical envelope to establish a relatively independent compartment that ensures genetic integrity and complex regulation of gene transcription. Also, the nucleus has been proposed to serve as a mechano-transducer of biological cues such as tissue damage-induced inflammatory signals^1^. Despite that the size and morphology of the nucleus vary in different types of cells, it is believed that the ratio of nuclear to cellular volume (referred to as the karyoplasmic ratio) remains constant^2^. It has been relatively well characterized regarding the breakdown and reconstitution of the nuclear envelope during cell cycle^3,4^. However, it remains poorly understood regarding potential changes of nuclear size and mechanics in a context of functional reprogramming of the cell. In particular, it is not clear whether the nucleus undergoes active remodeling for functional reprogramming in immune cells, a large class of cells that are usually sensitive to external or environmental stimuli for specific type of activation and function.

Macrophages play essential roles in both immune responses and tissue homeostasis in a manner depending on environmental cues^5^. Roughly, macrophages can be classified into two functional types termed M1 (proinflammatory or classically activated) and M2 (anti-inflammatory or alternatively activated). M1 macrophages can produce proinflammatory cytokines, such as IL-6, IL-12, IL-23, and TNFα, which inhibit the proliferation of surrounding cells and damage contiguous tissue. In this way, M1 macrophages play a key role in the inflammatory response and antitumor immunity. By contrast, M2 macrophages mainly resolve inflammation and promote wound healing and tissue repair^6^. The M1 *versus* M2 polarization of macrophage is a tightly controlled process, dysregulation of which has extensively associated with various inflammatory diseases and tumor progression^7^. How the plastic functions of macrophages are tailored to meet the needs of various physiological and pathological settings have attracted tremendous attention, but to date remain incompletely understood especially from an angle of mechanical regulation of the nucleus.

The evolutionarily conserved LINC (linker of the nucleoskeleton to the cytoskeleton) complexes, which are primarily composed of proteins containing SUN and KASH domains, may transduce mechanical force across the nuclear envelope^8,9^. Located on the inner nuclear envelope membrane, SUN domain proteins comprise an N-terminal nucleoplasmic domain, a transmembrane segment and a C-terminal SUN domain^9–12^. The nucleoplasmic domain of SUN proteins interacts with lamina and/or chromatin binding proteins, while the SUN domain protrudes into the lumen of the nuclear envelope to interact with KASH proteins in a trimeric fashion^13–17^. Defects in SUN proteins have been linked to human diseases including laminopathies, ataxia, progeria, lissencephaly, and cancer^18–21^.

Here, we report a SUN1/2-mediated mechanical remodeling phenomena of the nucleus with functional influence in macrophages. In response to inflammatory stimuli, the protein levels of SUN1/2 decrease to shrink and soften the nucleus, which thereby alters chromatin accessibility and gene transcription for M1 polarization of macrophages. Depletion of SUN1/2 strongly promotes M1 activation of macrophages in vitro and in vivo, leading to enhanced inflammation and antitumor immunity.

## Results

### Polarization signals induce mechanical alterations of the nucleus in macrophages

**Fig. 1:**
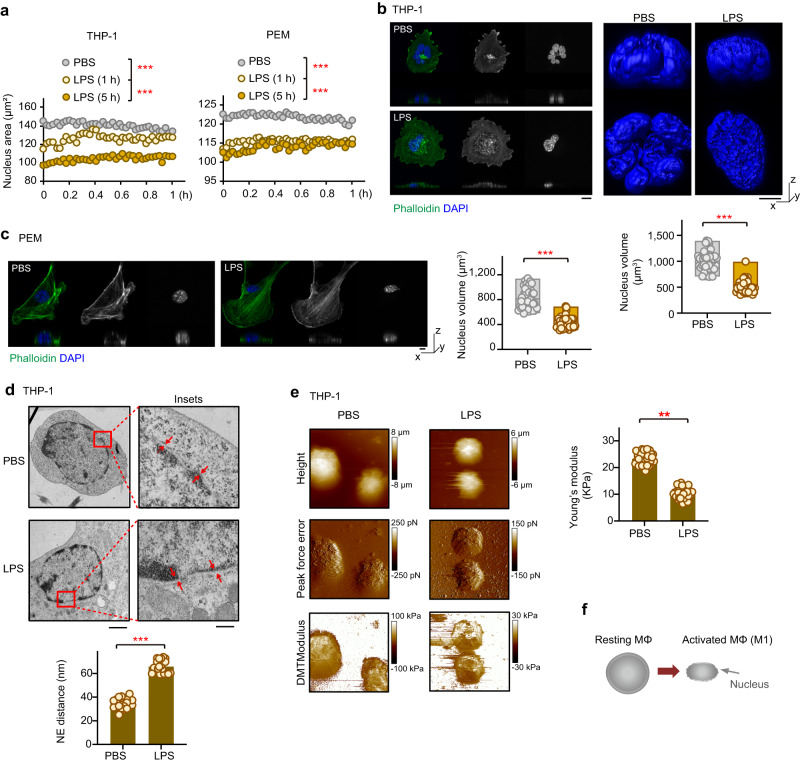
Morphological and mechanical remodeling of the nucleus in macrophages upon LPS stimulation. **a** The average size of the THP-1-derived macrophage or PEM nuclei after LPS challenge. THP-1-derived macrophages and PEMs were treated with 100 ng/ml LPS for indicated time periods. The cells were then washed with PBS and placed in normal culture condition. After live staining with Hoechst 33,342, the nuclei were observed every 90 s for 1 h using Opera High Content Screening Platform. For each time point, 5000 nuclei were measured to obtain the indicated average size. The x-axis represents hours after the LPS challenge. Fluorescence microscopy of Phalloidin and DAP in THP-1-derived macrophages (**b**, 40 cells per group) or PEMs (**c**, *n* = 48 cells per group) after LPS stimulation. The nuclear volume was quantified. In the box plots, the center line corresponds to the median and box corresponds to the interquartile range (IQR). Each dot represents one cell. Scale bar, 5 μm. **d** Nuclear envelope spacing determined by scanning electron microscopy. The nuclear envelope spacing was highlighted by red arrows and quantified as NE distance (*n* = 35 cells per group). Main image scale bar, 1 μm. Inset image scale bar, 0.2 μm. **e** Atomic force microscopy images of nuclei separated from LPS-treated macrophages and from controls (*n* = 20 cells per group). **f** A schematic model showing how the nucleus changes in size and shape during macrophage polarization. Two-sided Tukey post-hoc test was used to compared differences between groups after One-way ANOVA (**a**). Data were presented as mean ± SD (**d**,**e**). Two-sided unpaired student’s *t* test were used (**b**–**e**). **p < 0.01; ***p < 0.001 in comparison with control group. See also Supplementary Fig. 1.

As a potent activator of macrophages, lipopolysaccharide (LPS) can induce morphological alterations such as cell spreading, polymerization of cytoskeletal actin filaments, and the formation of filopodia, lamellipodia, and membrane ruffles^22^. To explore potential morphological and mechanical alterations of the nucleus, and to assess the functional importance of these alterations during polarization, we treated macrophages derived from THP-1, a human leukemia monocytic cell line, with LPS and stained their cellular DNA with Hoechst 33342. Over the course of 5 h of LPS challenge, the average area of the nuclei of the THP-1-derived macrophages decreased by ~25% as measured by high-content screening microplate imaging (Fig. 1a, *left*). Moreover, we also observed this phenomenon in mouse peritoneal elicited macrophages (PEMs) (Fig. 1a, *right*). These results were further confirmed by observations using fluorescent microscopy with DAPI staining (Supplementary Fig. 1).

Concomitant with the reduced area of the nuclei in LPS-treated macrophages, 3D confocal imaging showed significant decreases in the volumes of the nuclei of both THP-1-derived macrophages and PEMs upon LPS treatment (Fig. 1b, c, Supplementary Movie 1,2). We also used human bone marrow-derived macrophages (hBMs) to examine morphological changes of macrophages upon LPS treatment (Supplementary Fig. 1b). Our results showed that LPS treatment also led to a significant decrease in the average area and volume of nuclei of hBMs (Supplementary Fig. 1b). These results showed that the sizes of the nuclei become smaller in macrophages upon LPS treatment.

Next, we went on to investigate the underlying mechanisms for the LPS-induced shrinkage of the nucleus in macrophage. To this end, we examined the morphology of nuclear envelope, a double membrane structure. Our electron microscopy confirmed the LPS-induced shrinkage effect on the nucleus (Fig. 1d). Counterintuitively, however, LPS stimulation induced an ~50% wider nuclear envelope spacing (NE distance), namely the distance between the outer and inner membranes of the nuclear envelope (Fig. 1d). These results indicated that LPS-treatment may alter the mechanical nature of the nucleus in macrophage.

To detect possible mechanical alterations of the nucleus in LPS-treated macrophage, we separated nuclei from THP-1-derived macrophages treated with LPS or with PBS as a control. The nuclei were then subjected to analysis by using atomic force microscopy (AFM) with conical tips. When the tips approach and retract from the nuclei, peak force was detected to reflect the stiffness of nuclear matrix. As a measure of the stiffness of an elastic material, the average Young’s modulus can be calculated by fitting the contact region of the retract curve using the Derjaguin–Muller–Toporov model. As shown in Fig. 1e, the highest parts of the nuclei were decreased in LPS-treated cells, consistent with the above observed shrinkage effect. Moreover, the nuclear stiffness was significantly reduced after LPS stimulation (Fig. 1e). These observations indicated that LPS treatment could promote nuclear softening that would force the nucleus to deform dramatically, a feature that may facilitate macrophage migrating through narrow stromal environment to exert their functions.

In the presence of inflammatory stimuli and danger signals (LPS and IFN-γ), macrophages polarize toward an M1 state and release reactive oxygen species and inflammatory cytokines to fight pathogens, while a wound-healing environment (IL-4/IL-13) promotes polarization toward an M2 phenotype and leads to cellular processes that facilitate tissue repair. Inducible nitric oxide synthase (iNOS) and arginase-1 are well-established markers of M1 and M2 phenotypes, respectively^23^. To further characterize the nuclear mechanics associated with macrophage polarization, we compared the sizes of M1 and M2 nuclei to that of M0, a state before polarization. The polarization states were confirmed by expression of iNOS and Arginase-1, as well as their cellular morphology. In response to the M1 polarization signal LPS and IFN-γ, the nuclei of PEM cells shrank significantly, as was observed for LPS-induced activation (Supplementary Fig. 1c). In contrast, addition of IL-4, which stimulates M2 polarization, led to a marginal and statistically insignificant increase in the size of the nucleus. Clearly, the size of the nucleus in the M1 state was found to be substantially smaller than that in the M2 state. These observations revealed differential alterations of the nuclei dependent on the distinct polarization state of the macrophage.

GM-CSF and M-CSF are key factors involved in the differentiation of monocytes to macrophages^24^. Human GM-CSF can polarize monocytes towards the M1 macrophage subtype with a pro-inflammatory cytokine profile (e.g., TNFα, IL-6) (M1-like), while treatment of monocytes with M-CSF produces an anti-inflammatory cytokine profile (e.g., IL-10) similar to M2 macrophage (M2-like). To investigate the nuclear mechanics in monocyte differentiation, we treated bone marrow-derived monocytes with GM-CSF and M-CSF, respectively. Similar to the case of M1-M2 polarization, the size of the M1-like nucleus was found to be significantly smaller than that of the M2-like nucleus over the course of differentiation (Supplementary Fig. 1d).

Together, these results indicate that the nucleus of a macrophage undergoes morphological (shrinkage in size and widening in nuclear envelope spacing) and mechanical (softening and less elastic) changes during its M1 polarization (Fig. 1f).

### LPS stimulation decreases the levels of nuclear envelope protein SUN1/2 in macrophages

**Fig. 2:**
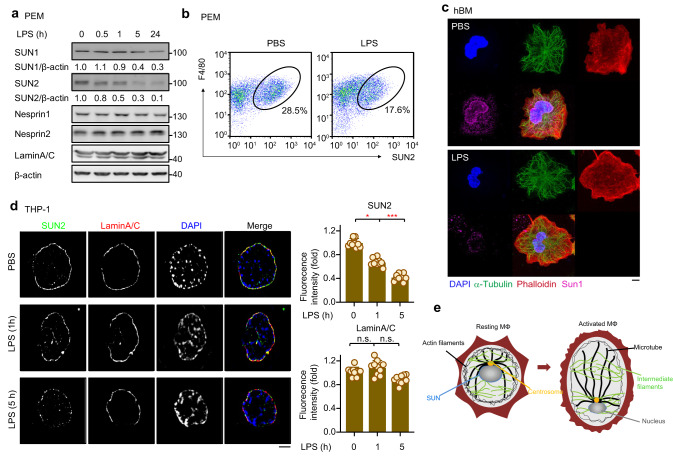
Decreases of the SUN1/2 protein levels in response to inflammatory cues. **a** Expression levels of the indicated proteins in PEMs that were challenged with LPS for various periods of time. **b** Flow cytometry analysis of SUN2 expression in F4/80^+^ PEMs after LPS stimulation for 5 h. **c** Immunofluorescence analysis of α-Tubulin (green), Phalloidin (Red), Sun1 (purple) and DAPI (blue) in hBM after LPS stimulation. LPS, 100 ng/ml. Scale bar, 5 μm. **d** Fluorescence microscopy of SUN2 and LaminA/C in LPS-stimulated macrophages (*n* = 10 cells per group). **e** A schematic model of SUN-mediated alterations to the structure of the macrophage during LPS-induced activation. Representative of 2 independent experiments (**a**, **c**). Data were presented as means ± SD (**d**). Two-sided Tukey post-hoc test was used to compared differences between groups after One-way ANOVA (**d**). *p < 0.05; ***p < 0.001, n.s no significance (*p* > 0.05) in comparison with control group. See also Supplementary Fig. 2.

To interpret the LPS-induced remodeling of the nucleus in macrophages, in particular the widened nuclear spacing, we hypothesized that M1 polarization signals like LPS may initially alter the levels of certain nuclear envelope proteins and thereby alter the mechanical property and the size of the nucleus. To test this possibility, we examined the LPS-induced expression patterns of several confirmed nuclear envelope proteins including LaminA/C, SUN1/2 and Nesprin1/2. The antibody that we produced to detect human SUN2 was first evaluated by western blotting and immunofluorescence (Supplementary Fig. 2a, b). A similar decrease of SUN2 levels after 1 or 5 h of LPS treatment was also observed for PEMs (Fig. 2a). Consistent with these results, flow cytometry analysis also showed the SUN2 protein levels to be significantly lower in LPS-treated F4/80-positive PEMs than in PBS-treated cells (Fig. 2b, Supplementary Fig. 2b). Subsequent confocal imaging showed that LPS treatment induced not only an apparent reduction of the size of the nucleus, but also a markedly decreased fluorescence intensity of SUN proteins (but not of LaminA/C) in hBM, THP-1-derived macrophages and PEMs (Fig. 2c, d, Supplementary Fig. 2c). Together with the previously reported mechanical roles of LINC complexes^13,14^, these observations indicate SUN1/2 proteins as molecular mediators in LPS-induced morphological and mechanical remodeling of the nucleus in macrophage (Fig. 2e).

### LPS promotes degradation of SUN1/2 proteins via βTrCP-mediated ubiquitination

**Fig. 3:**
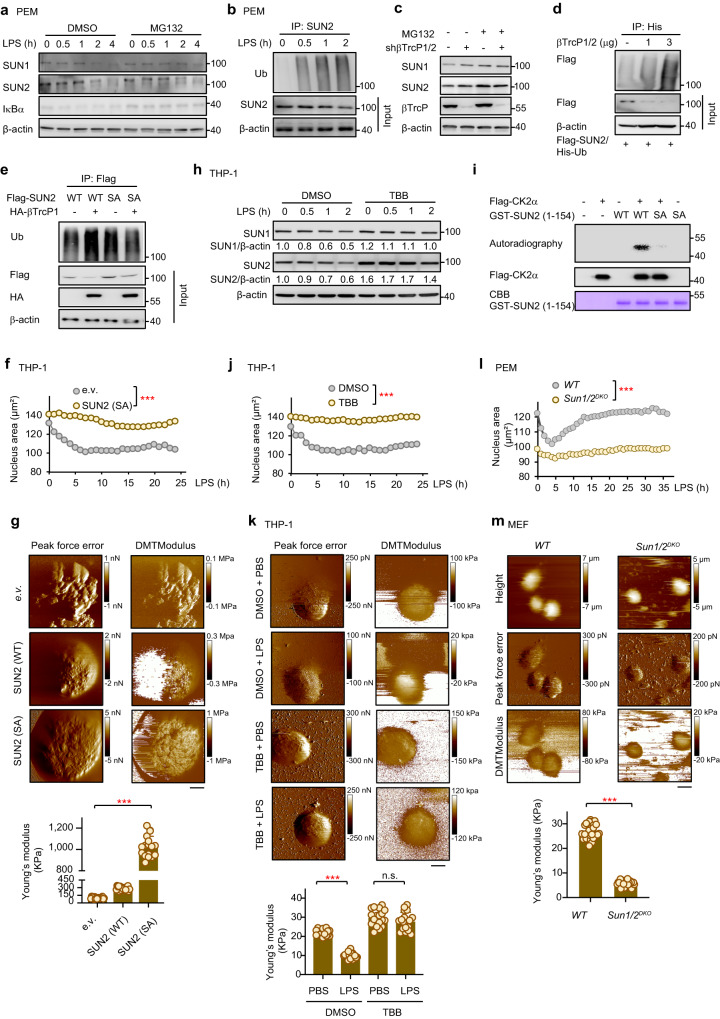
A CK2-βTrCP axis regulates SUN1/2 degradation to mediate LPS-induced remodeling of the nucleus in macrophages. **a** Protein levels of SUN1/2 in LPS-stimulated PEMs after being treated with MG132. **b** Ubiquitination of SUN2 in PEMs upon LPS stimulation. **c** Immunoblotting analysis of SUN1/2 in βTrCP1/2-knockdown 293 T cells after being treated with MG132. **d** Ubiquitination of SUN2 after transfection of 293 T cells with βTrCP. **e** Ubiquitination of wildtype SUN2 and its SA mutant in which the βTrCP-binding motif was disrupted. WT, wildtype; SA, SUN2 (S131A/S132A/S136A). **f** The average size of the nucleus of THP-1-derived macrophages stably expressing the SUN2 SA mutant. *e.v*., empty vector; SA, SUN2 (S131A/S132A/S136A), same below. **g** AFM images of nuclei from HEK293FT cells after transfection with SUN (WT) or the SUN2 SA mutant (*n* = 18 nuclei per group). Scale bar, 5 μm. **h** Immunoblotting of SUN1/2 in LPS-stimulated macrophages after being treated with TBB, a CK2 inhibitor. **i** In vitro kinase assay for CK2 using purified recombinant proteins of SUN2 (WT) and SUN2 (SA) as substrates. **j** Average size of THP-1-derived macrophage nuclei after treatment with TBB. **k** AFM images of nuclei from THP-1-derived macrophages treated with TBB (*n* = 18 nuclei per group). Scale bar, 5 μm. **l** Average size of SUN1/2 (*WT*) and *Sun1/2*^*DKO*^ PEMs nuclei after treatment with LPS. **m** Mechanical properties of nuclei in SUN1/2 (*WT*) and *Sun1/2*^*DKO*^ MEFs after treatment with LPS (*n* = 33 nuclei per group). Scale bar, 5 μm. Representative of 2 independent experiments (**a**–**e**, **h**, **i**). Data were presented as means ± SD (**f**, **j**, **k**). Two-sided unpaired student’s *t* test was used to compare difference between two groups (**f**, **j**, **k**). *p < 0.05; **p < 0.01; ***p < 0.001, n.s. no significance (*p* > 0.05) in comparison with control group. See also Supplementary Fig. 3.

We then investigated the mechanism by which SUN1/2 protein levels were decreased in macrophages in response to LPS stimulation. We first examined whether SUN1/2 downregulation is dependent on the proteasomal pathway. Treatment of PEMs with the proteasomal inhibitor MG132 significantly blocked the LPS-induced decrease of SUN1/2 proteins (Fig. 3a), suggesting that LPS regulates SUN1/2 via protein degradation. Consistent with this result, LPS treatment progressively enhanced ubiquitination of SUN2 (Fig. 3b). Meanwhile, no significant change of SUN1/2 transcription was detected in LPS-treated cells (Supplementary Fig. 3a). Together, these data indicate that LPS induces downregulation of SUN proteins by promoting their ubiquitination and proteasomal degradation.

Next, we set out to determine which E3 ubiquitin ligase accounts for the degradation of SUN proteins. Our mass spectrometry analysis (Supplementary Fig. 3b) and immunoprecipitation assay (Fig. 3c) revealed βTrCP1/2 to be a potential interaction partner of SUN1/2. To validate the functional relevance of βTrCP1/2 in the degradation of SUN proteins, we transfected 293 T cells with short hairpin RNA targeting βTrCP1/2 (shβTrCP1/2) and then performed an immunoblotting assay. As shown in Fig. 3c, knockdown of βTrCP1/2 obviously enhanced the SUN1/2 protein levels while MG132 treatment blocked shβTrCP1/2-induced accumulation of SUN1/2. Consistent with this observation, co-transfection of βTrCP1/2 enhanced the ubiquitination of SUN2 in a dose-dependent manner and decreased its protein level (Fig. 3d). Together, these results indicate that βTrCP1/2 act as an E3 ligase of SUN proteins.

Sequence analysis further revealed “SSSGYSSSEDD” as a potential βTrCP1/2-binding motif that is conserved among SUN proteins from various mammalian species (Supplementary Fig. 3d). Mass spectrometry analysis identified serine 136 in the motif to be a candidate phosphorylation site (Supplementary Fig. 3e). A modeled structure of βTrCP1/2 in complex with this motif also suggests that βTrCP1/2 could bind SUN proteins in a classical phosphorylation-dependent manner^25^, and that SUN serines 131 and 132 could also be involved in binding βTrCP1/2 (Supplementary Fig. 3f). To determine whether the putative βTrCP1/2 recognition motif is responsible for βTrCP1/2-SUN interaction, we substituted serines 131/132/136 of wildtype SUN (WT) with alanines to create a SUN (SA) mutant, which would prevent phosphorylation and subsequent βTrCP1/2-binding and ubiquitination of SUN proteins. Indeed, the protein level of SUN2 (SA) was higher than that of SUN2 (WT) in THP-1-derived macrophages transfected with similar amount of plasmids of wildtype or mutant *SUN2* (Supplementary Fig. 3g). Moreover, overexpression of βTrCP1 significantly increased the ubiquitination of wildtype SUN2 but not of its SA mutant (Fig. 3e). Consistently, immunofluorescent assay further showed that overexpressed SUN2 (SA) was able to locate on the nuclear envelope, and that SUN2 (SA) mutant but not SUN2 (WT) was more resistant to LPS-induced degradation (Supplementary Fig. 3h, i).

### CK2 phosphorylates SUN1/2 for their binding with βTrCP and LPS induced degradation

Since phosphorylation of SUN proteins is required for recognition by βTrCP during LPS-induced degradation, we further determined the specific kinase accounting for such phosphorylation. Among the candidate protein kinases indicated by Motif Scan^26^, CK2 was predicted to phosphorylate SUN2 at S136 based on the consensus sequence S/T-X-X-D/E. Meanwhile, our mass spectrometry also showed a potential interaction between SUN2 and CK2 (Supplementary Fig. 3j). Based on these results and a previous report that activation of CK2 can be induced by LPS in macrophages^27^, we reasoned that CK2 activity may be important for LPS-induced decrease of SUN1/2 protein levels. To test this possibility, we used 4,5,6,7-tetrabromobenzotriazole (TBB), a CK2-specific inhibitor, to treat THP-1-derived macrophages in the presence of LPS. Our results showed that TBB but not DMSO blocked LPS-induced decrease of SUN protein levels (Fig. 3h), indicating that CK2 activity is required for LPS-induced degradation of SUN proteins. Moreover, this observation was further confirmed by an in vitro kinase assay showing that CK2 could directly phosphorylate purified recombinant wildtype SUN2 protein (Fig. 3i). Meanwhile, CK2 failed to phosphorylate the SA mutant protein of SUN2, suggesting that CK2 phosphorylates SUN2 at the βTrCP1/2-binding motif. Together, these results demonstrated that the kinase CK2 phosphorylates SUN proteins to facilitate their binding with βTrCP during LPS-induced degradation.

### SUN1/2 mediate LPS-induced morphological and mechanical remodeling of the nucleus

Based on our findings of the nucleus and the nuclear envelope proteins in LPS-treated macrophages, we hypothesized that SUN1/2 proteins mediate LPS-induced morphological and mechanical remodeling of the nucleus. To test whether expression levels of SUN proteins indeed regulate the mechanics of the nucleus, we transfected THP-1 cells with the SUN2 SA mutant that is resistant to βTrCP1/2-mediated ubiquitination and degradation. Unlike those of control cells, the average area of the nuclei of the cells expressing the SUN2 SA mutant was not affected by LPS stimulation (Fig. 3f, Supplementary Fig. 3h, i). Furthermore, the elasticity and stiffness of the nuclei of cells transfected with the SUN2 SA mutant were significantly higher than those in cells transfected with an empty vector or wildtype SUN2 (Fig. 3g). Together, these results indicate that SUN protein levels modulate LPS-induced morphological and mechanical alterations of the nucleus in macrophages.

Since SUN protein levels are regulated by CK2-βTrcP-dependent proteasomal degradation, we also tested the potential effect of CK2 on LPS-induced morphological and mechanical alterations of the nucleus. To this end, we used TBB to block the kinase activity of CK2. Indeed, similar to the case of the SUN2 SA mutant, the average size of the nuclei of TBB-treated cells was not affected by LPS stimulation (Fig. 3j). The elasticity and stiffness of these nuclei were also not influenced by LPS in the presence of TBB (Fig. 3k).

To further corroborate the effect of SUN protein levels on the size and mechanics of the nucleus, we examined the nuclei from cells in which the expressions of SUN1/2 were silenced. As shown in Fig. 3l, the nuclei of *Sun1/2*^*DKO*^ PEMs were significantly smaller than those of wildtype cells. Moreover, the size of the nucleus was no longer influenced by LPS stimulation in cells lacking SUN proteins (Fig. 3l). Consistent with these results, the elasticity and stiffness of nuclei of *Sun1/2*^*DKO*^ mouse embryonic fibroblasts (MEFs) were much lower than those of wildtype cells (Fig. 3m). Given that LINC complexes mechanically connect ECM and cytoskeleton to the nucleus^8,9^, we reasoned that the stiffness changes of the nucleus may be coupled with that of the cell surface. To test this possibility, we first disrupted actin cytoskeleton with cytochalasin D (Cyto D), an event which would result in loss of LINC-mediated tension, leading to decreased nuclear size and lower cellular stiffness. Normally, PEMs formed filopodia containing tight bundles of long actin filaments covered with cell membrane; yet Cyto D treatment reduced filopodia formation (Supplementary Fig. 3k). Moreover, Cyto D treatment decreased the nuclear sizes of PEMs (Supplementary Fig. 3k). Importantly, Cyto D treatment also decreased the cell membrane stiffness as measured by AFM (Supplementary Fig. 3l). Furthermore, compared to wild-type PEMs, Sun1/2-deficient PEMs exhibited lower average cell membrane stiffness as measured by AFM (Supplementary Fig. 3m), mimicking the Cyto D-induced phenotype.

Taken together, these results demonstrated a key role for SUN1/2 in mediating LPS-induced mechanical alterations of the nucleus, and such alterations can be transmitted to the cell membrane via LINC complexes.

### Deficiency of SUN1/2 alters chromatin conformation and enhances chromatin accessibility

**Fig. 4:**
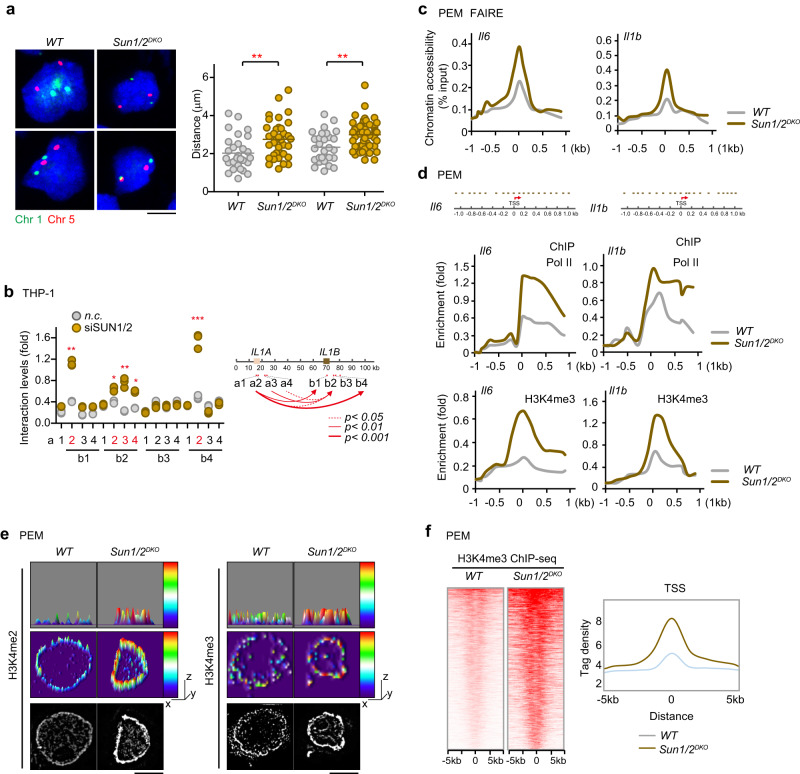
Depletion of SUN1/2 promotes chromatin openness at *Il6* and *Il1b* loci. **a** FISH analysis in *WT* and *Sun1/2*^*DKO*^ PEMs by using Chromosome 1 and 5 probes (*n* = 28 cells per group). Scale bar, 5 μm. **b** 3 C assay to detect interaction levels (relative positioning) between sites from *IL1A* and *IL1B* in the THP-1-derived macrophages (*n* = 3 biological replications per group). **c** FAIRE assay to detect accessibility and openness of chromatin at *Il6* and *Il1b* loci in in *WT* and *Sun1/2*^*DKO*^ PEMs. **d** ChIP assay to analyze the enrichment of Pol II and H3K4me3 on *Il6* and *Il1b* loci in *WT* and *Sun1/2*^*DKO*^ PEMs. **e** H3K4me2 and H3K4me3 staining in *WT* and *Sun1/2*^*DKO*^ PEMs. Scale bar, 5 μm. **f** ChIP-Seq of H3K4me3 in *WT* and *Sun1/2*^*DKO*^ PEMs. Heat maps showing the distributions of H3K4me3 within 10 kb of the TSSs. Composite H3K4me3 distribution profiles within 10 kb of the TSSs. ChIP assays were performed with anti- H3K4me3 antibodies in PEMs, followed by ChIP-Seq. *p < 0.05; **p < 0.01; ***p < 0.001 in comparison with control group. Two-sided Tukey post-hoc test was used to compared differences between groups after One-way ANOVA (**a**, **b**). Representative of 2 independent experiments (**e**). See also Supplementary Fig. 4.

Since the nuclear envelope proteins SUN1/2 mediate LPS-induced nuclear remodeling during M1 polarization of macrophages, we hypothesized SUN1/2 may play a functional role in regulating chromatin accessibility and gene transcription. To this end, we first performed fluorescence in situ hybridization (FISH) assay and examined homologous chromosome arrangement. The result showed that knockout of SUN1/2 in PEMs induced significant increases of distances between homologous chromosomes 1 and 5 (Fig. 4a), indicating a regulatory role for SUN1/2 in chromatin conformation. Since the two principal members of the IL-1 cytokine family, *IL1A* and *IL1B*, are encoded within 30 kb of each other, within the peak of the interval on Chr2 (129 Mb), and thus made the regulatory regions of *IL1A* and *IL1B* come in close proximity upon LPS stimulation^28^, we next performed chromatin conformation capture (3 C) analysis using multiple primers targeting *IL1A* and *IL1B* in THP-1-derived macrophages (Fig. 4b). By analyzing the positive interactions of different fragments of *IL1A* and *IL1B*, it appears that the interaction is maximal at the promoter regions (a2-b2) to the intragenic fragments (a3-b1). Confirming their regulatory effect on chromatin conformation, knockdown of SUN1/2 substantially enhanced the interaction levels between multiple sites probed, especially those between a2 and b4, a2 and b1, a3 and b2.

To assess the role of SUN1/2 in maintaining a permissive chromatin status for inflammation-related genes, we performed FAIRE (Formaldehyde-Assisted Isolation of Regulatory Elements) assay in PEMs. Compared with the transcription start site (TSS)-distal regions, TSS-proximal regions of *Il6* and *Il1b* were highly enriched in FAIRE-DNA samples from wildtype PEMs (Fig. 4c), indicative of an open, and active chromatin conformation. Silencing of SUN1/2 expression further substantially increased the accessibility at the *Il6* and *Il1b* locus (Fig. 4c). Again, these data indicate that SUN1/2 regulate chromatin conformation.

To further corroborate the impact of SUN proteins on the openness and active state of chromatin, we detected the recruitment of DNA polymerase II (Pol II) to the TSS of *Il6* and *Il1b* genes. Knockout of SUN1/2 in PEMs greatly promoted the enrichment of Pol II on the TSS and ~1 kb downstream region of *Il6* and *Il1b* (Fig. 4d, upper). Meanwhile, H3K4me3, typically found in the promoter region of genes and thought as a marker associated with an open chromatin state and active gene transcription, was dramatically enriched in these regions upon silencing of SUN1/2 (Fig. 4d, lower). As a negative control, H3K27me3, a repressive marker of gene transcription, was not significantly altered (Supplementary Fig. 4), consistent with previous studies^29,30^. Subsequently, we assessed globally the impact of SUN1/2 on the epigenetic status of chromatin. Immunostaining of PEMs with antibodies specifically recognizing H3K4me2 and H3K4me3 respectively, followed by imaging and reconstitution revealed much higher levels of both H3K4me2 and H3K4me3 in SUN1/2 deficient nucleus than in wildtype nucleus (Fig. 4e). Our chromatin immunoprecipitation sequencing (ChIP-seq) identified 1,102 and 1,360 peaks of H3K4me3 in *WT* and *Sun1/2*^*DKO*^ PEMs (Fig. 4f), respectively, with 803 overlapping peaks (Supplementary Fig. 4b). Moreover, the peaks of H3K4me3 were greatly attenuated in *Sun1/2*^*DKO*^ PEMs under resting conditions (Fig. 4f), indicating a genome-wide transcriptional activation effect after SUN ablation. Further KEGG analysis of the upregulated peaks (Top 200) revealed that inflammatory pathway genes (*Il1b*, *Tnfa*, *Nos2*), ECM remodeling genes (*Actin2*, *Adamts15b*, *Mmp25*) and endocytosis genes (*Rab11a*, *Rab5*) were enriched (Supplementary Fig. 4c), indicating an open chromatin state in *Sun1/2*^*DKO*^ PEMs.

Together, these results revealed that decrease of SUN proteins alters chromatin conformation to promote chromatin openness and accessibility.

### Deficiency of SUN1/2 facilitates nuclear translocation of the transcription factor p65

To probe possible impact of SUN proteins on LPS signaling upstream events, we measured NF-κB activation induced by major components of TLR4 pathway in HEK293T cells transfected with an NF-κB luciferase reporter. As shown in Supplementary Fig. 4d, knockdown of SUN1/2 promoted MyD88-, IRAK1-, IKKβ-, TRAF6- or p65-induced NF-κB activation. Since SUN proteins are mainly located on the inner nuclear membrane, we reasoned that SUN1/2 may regulate TLR4 signaling by interaction with the downstream transcriptional factor p65. Indeed, we performed super-resolution immunofluorescence imaging, and observed a clear signal of co-localization between SUN2 and p65 (Supplementary Fig. 4e). Given the fact that p65 shuttles between the cytoplasm and the nucleus in a signal-dependent manner, we reasoned that SUN1/2 proteins might affect the nuclear translocation of p65. To test this possibility, we detected localization of p65 in *Sun1/2*^*DKO*^ PEMs with or without LPS treatment. As expected, LPS stimulation significantly induced nuclear translocation of p65 (Supplementary Fig. 4h). Importantly, depletion of SUN1/2 also resulted in nuclear translocation of p65 even in the absence of LPS (Supplementary Fig. 4f). Moreover, LPS treatment of *Sun1/2*^*DKO*^ cells resulted in much stronger nuclear translocation of p65 (Supplementary Fig. 4f). Taken together, these results indicate that LPS-induced reduction of SUN protein levels promotes p65 nuclear translocation.

### Deficiency of SUN1/2 promotes gene transcription for inflammation and immune responses

To determine the functional consequence of SUN1/2 regulation of chromatin conformation and accessibility, we used whole-transcriptome microarrays to detect the global impact of SUN1/2 on gene transcription in LPS-treated macrophages. We found that 533 and 686 of genes exhibited significantly up-regulation and down-regulation, respectively in SUN1/2-knockdown macrophages relative to their expressions in control cells, indicating a critical role of SUN1/2 in the regulation of these genes' transcription (Supplementary Fig. 5a). Subsequent gene-ontology enrichment analysis revealed that the affected genes are associated with 659 biological processes, 717 cellular components and 690 molecular functions. Among these genes, 126 encoded factors involved in innate immune responses, 119 genes in cellular protein metabolic process and 64 genes in protein transport, indicating that SUN1/2 proteins function as key modulators not only in immune response, but also in several other biological processes, such as endocytosis, and metabolic process. Further KEGG analysis divided the candidate genes into 41 signaling pathways, among which the top 25 enriched pathways were listed with enrichment factor. The pathways involved in inflammation and immune responses were: Type I interferon signaling pathway, TNF signaling pathway, RIG − I−like receptor signaling pathway, Toll-like receptor signaling pathway, Cytokine-mediated signaling pathway, NOD−like receptor signaling pathway, Jak−STAT signaling pathway, chemokine signaling pathway (Supplementary Fig. 5b). Together, these results indicated that SUN proteins modulate the transcription of many genes involved in multiple inflammatory pathways.

### SUN1/2 regulate LPS-induced gene expression in a mechanosensitive manner

**Fig. 5:**
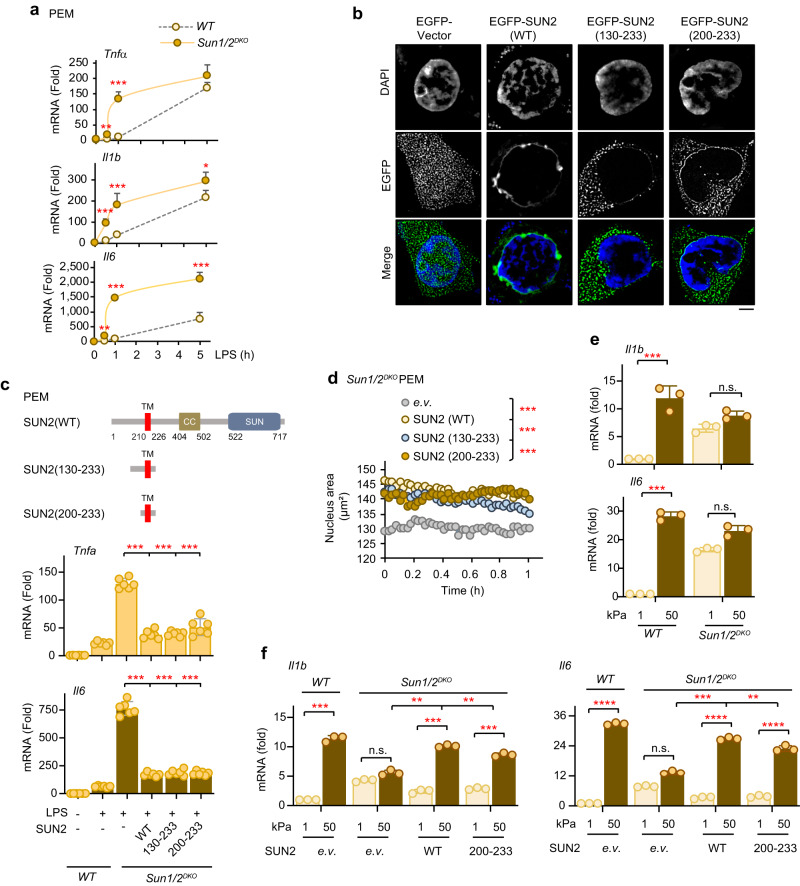
Depletion of SUN1/2 enhances inflammatory cytokine production in a mechanical manner. **a** Transcription of cytokines in *WT* and *Sun1/2*^*DKO*^ PEMs after being challenged with LPS (*n* = 3 biological replications per group). Data were presented as means+SD. **b** Immunofluorescent images of EGFP in 293FT cells after transfected with the indicated plasmids. Wild type (WT) PEMs were presented for comparison. SUN truncations (amino acids 130–233, amino acids 200–233) were presumably disabled for binding to both Nesprins and Lamins. Scale bar, 5 μm. **c** Rescue of the SUN-mediated suppression of the LPS-induced transcription of *TNFα* and *Il6* in *Sun1/2*^*DKO*^ PEMs by transfection of wildtype SUN2 and its truncations (*n* = 6 biological replications per group). **d** Average size of SUN1/2 (WT) and its truncations-transfected PEMs nuclei after treatment with LPS. **e** QPCR assay showing *Il1b* and *Il6* transcription in the indicated PEMs using two collagen-coated polyacrylamide hydrogels (*n* = 3 biological replications per group). **f** Rescue assay showing *Il1b* and *Il6* transcription in the indicated PEMs using collagen-coated polyacrylamide hydrogels mimicking different ECM stiffness (1 and 50 kPa, *n* = 3 biological replications per group). WT, wildtype; *e.v*., empty vector. Representative of 2 independent experiments (**b**). Data were presented as means ± SD (**c**, **e**, **f**). Two-sided Tukey post-hoc test was used to compared differences between groups after One-way ANOVA (**a**, **c**–**f**). *p < 0.05; **p < 0.01; ***p < 0.001, ****p < 0.0001, in comparison with control group. See also Supplementary Fig. 5.

To better study the functional role of SUN1/2-mediated nuclear remodeling, we examined the mRNA levels of proinflammatory cytokines (*Tnfα*, *Il1b*, *Il6*) in wildtype and SUN1/2-knockout PEMs treated with LPS. Consistent with the above observations in chromatin conformation and gene expression, deficiency of SUN1/2 dramatically promoted LPS-induced production of the cytokines *Tnfα*, *Il1b*, and *Il6* (Fig. 5a). A subsequent rescue experiment further revealed that transfection of wildtype SUN2 into *Sun1/2*^*DKO*^ PEMs inhibited LPS-induced *Il6* transcription, and expression of the SUN2 SA mutant that is resistant to LPS-induced degradation inhibited *Il6* transcription to an even larger extent (Supplementary Fig. 5c). Together, these results demonstrated that SUN proteins indeed regulate LPS-induced gene transcription.

Next, we mapped the specific domain(s) required for SUN1/2 regulation of LPS-induced gene expression. SUN1/2 proteins contain an N-terminal nucleoplasmic domain that interacts with the nuclear lamina and chromatin binding proteins, a transmembrane segment that anchors them to the inner nuclear membrane, and a conserved C-terminal SUN domain that protrudes into the lumen of the nuclear envelope to interact with outer nuclear membrane proteins^9,10,12–14,17^ (Supplementary Fig. 5c). We then transfected various truncation mutants of SUN1/2 back into *Sun1/2*^*DKO*^ PEMs and examined their ability to rescue the inhibitory effect on LPS-induced gene expression when compared with wildtype SUN1/2. Notably, transfection of a SUN2 truncation mutant (DelTM) in which the transmembrane domain was deleted did not alter the amount of *Tnfα* and *Il6* mRNA produced in *Sun1/2*^*DKO*^ PEMs, suggesting an essential role for the transmembrane domain in the regulation of gene transcription (Supplementary Fig. 5c).

Surprisingly, transfection of *Sun1/2*^*DKO*^ PEMs with a SUN2 truncation mutant (the nucleoplasmic domain plus the transmembrane domain: amino acids 1–234) that could not bind to Nesprin inhibited the production of cytokines *Tnfα* and *Il6* as did wildtype SUN2, suggesting that binding to Nesprin is dispensable for SUN2 regulation of LPS-induced gene expression (Supplementary Fig. 5c, d). Moreover, transfection of *Sun1/2*^*DKO*^ PEMs with a SUN2 truncation mutant (the nucleoplasmic linker plus the transmembrane domain: amino acids 130–233) that would disable its binding to LaminA/C also inhibited the production of cytokines *Tnfα* and *Il6* as did wildtype SUN2, suggesting that binding to LaminA/C is dispensable too for SUN2 regulation of LPS-induced gene expression (Fig. 5b, c, Supplementary Fig. 5c).

Based on these observations and considering the roles of SUN1/2 in mediating LPS-induced nuclear remodeling, we speculated that SUN proteins' transmembrane domain alone was sufficient to mechanically alter the chromatin accessibility and thereby to exert the regulatory functions in LPS-induced gene expression. Indeed, transfection of *Sun1/2*^*DKO*^ PEMs with a SUN2 truncation mutant (the transmembrane domain: amino acids 200-233) readily inhibited the production of cytokines *Tnfα* and *Il6* as did wildtype SUN2 (Fig. 5b, c). Moreover, transfection of *Sun1/2*^*DKO*^ PEMs with a SUN2 truncation fragment containing the transmembrane domain (amino acids 130–233) or just the transmembrane domain alone (amino acids 200–233) recovered the average size and stiffness of the nuclei as did wildtype SUN2 (Fig. 5d, Supplementary Fig. 5e).

To further test whether SUN1/2 regulation of LPS-induced gene expression depends on SUN1/2-mediated remodeling of the nucleus, we used various concentrations of matrigel to mimic mechanical stress. To this end, the nuclei of PEMs were extracted and incubated in 3D matrigel. Consistent with observations in Fig. 5a, depletion of SUN1/2 markedly increased the transcriptions of proinflammatory cytokines *Il1b* and *Il6*; yet such effects were inhibited in a dose-dependent manner by applying higher concentration of matrigel mimicking a stiffer microenvironment (Supplementary Fig 5f). Moreover, the rescuing effects observed for transfection of *Sun1/2*^*DKO*^ PEMs with wildtype SUN2 or various truncation mutants (amino acids 130–233; amino acids 200–233) were overridden in terms of *Il6* transcription by applying the isolated nuclei onto a stiffer matrix (Supplementary Fig. 5g).

As the nucleus is mechanically connected to the cell surface via LINC complexes, we further examined SUN1/2 mechanoregulation of LPS signaling by manipulating the mechanical environment of the cell surface. To this end, we plated intact PEMs on two polyacrylamide hydrogels with different stiffness (1 and 50 kPa, respectively). Using RT-PCR analysis, we found a significant upregulation in the transcription of *Il1b* and *Il6* with high stiffness (50 kPa) compared to low stiffness (1 kPa) in wildtype PEMs (Fig. 5e). Ablation of Sun1/2 in PEMs further increased the transcription of *Il1b* and *Il6* with the stiffness of 1 kPa but not 50 kPa (Fig. 5e). Moreover, the stiffness-induced differential transcriptions of *Il1b* and *Il6* were diminished in *Sun1/2*^*DKO*^ PEMs (Fig. 5e). Importantly, back transfection of *Sun1/2*^*DKO*^ PEMs with wildtype SUN2 or just a SUN2 transmembrane domain (amino acids 200–233) that could not bind to either LaminA/C or Nesprin1 (Supplementary Fig. 5d) recovered the differential effect (Fig. 5f).

Together, these results indicate that SUN proteins regulate LPS-induced gene expression via mechanical alteration of the nucleus, and that the transmembrane domain is both required and sufficient for such alteration.

### Deficiency of SUN1/2 facilitates M1 polarization of macrophages

**Fig. 6:**
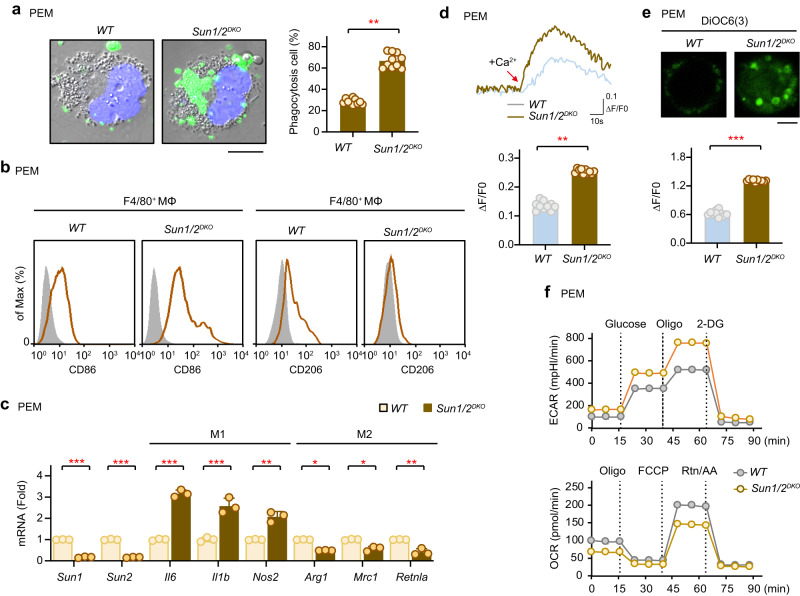
Depletion of SUN1/2 facilitates M1 polarization of macrophages. **a** Representative images showing phagocytosis of *WT* and *Sun1/2*^*DKO*^ PEMs (upper), and the quantified efficiency of phagocytosis calculated for 200 cells (lower). Scale bar, 1 μm. **b** Flow cytometry analysis of the macrophage-activation markers CD86 (M1) and CD206 (M2). The colored curves represent cell staining with CD86 or CD206 antibody, and the gray curve represents cell staining with a control antibody. **c** Transcriptional levels of M1 macrophage markers (*Il6, Il1b* and *Nos2*) and M2 macrophage markers (*Arg1*, *Mrc1* and *Retnla*) in *WT* and *Sun1/2*^*DKO*^ PEMs (*n* = 3 biological replications per group). **d** Ca^2+^ trace in *WT* and *Sun1/2*^*DKO*^ PEMs (*n* = 10 biological replications per group). Each circle depicts mean value of the fluorescence ratio (340/380) from 50 cells. Scale bar, 1 μm. **e** Mitochondrial integrity of *WT* and *Sun1/2*^*DKO*^ PEMs (*n* = 10 biological replications per group). PEMs were incubated with DiOC6 (40 nM) for 30 min. Each circle depicts mean value of the fluorescence ratio (340/380) from 50 cells. **f** Metabolic profiles (ECAR and OCR) of *WT* and *Sun1/2*^*DKO*^ PEMs. The vertical dotted lines represent the specific time points when the indicated agents were added. WT, wildtype. Data are presented as mean ± SD (**a**, **c**–**e**). Two-sided unpaired student’s *t* test was used to compare difference between two groups (**a**, **c**–**e**). *p < 0.05; **p < 0.01; ***p < 0.001 in comparison with control group. See also Supplementary Fig. 6.

To further assess the functional importance of SUN1/2-mediated nuclear remodeling and transcriptional regulation, we first examined the phagocytosis of PEMs. Depletion of SUN1/2 in PEMs markedly enhanced their phagocytic ability and promoted the efficiency of phagocytosis (to a level of ~60%) as compared to control cells (~32%) (Fig. 6a). Also, we assessed the potential effect of SUN1/2 on the mobility of macrophages using a Transwell chamber. The results showed that knockout of SUN1/2 in PEMs significantly enhanced their efficiency of migration and infiltration in vitro (Supplementary Fig. 6a).

Next, we examined the effect of SUN1/2 on M1 or M2 polarization of macrophages. Flow cytometry analysis showed that the proportion of M1 (CD86^+^) cells was highly increased in *Sun1/2*^*DKO*^ PEMs, whereas the percentage of M2 (CD206^+^) cells was obviously decreased by SUN1/2 knockout (Fig. 6b). Moreover, M2 polarization (F4/80^+^CD206^+^) of PEMs was significantly blocked by siRNA silencing of SUN1/2 (Supplementary Fig. 6b). Furthermore, we profiled the expression of key genes that mark commitment to M1 or M2 polarization in PEMs. Depletion of SUN1/2 substantially increased the expression of M1 markers including *Il6, Il1b* and *Nos2*, but reduced the expression of the M2 markers such as *Arg1* and *Mrc1* (mannose receptor CD206) and *Retnla* (Fig. 6c).

Given the importance of intracellular Ca^2+^ levels in macrophage function^31–33^, we went on to characterize Ca^2+^ signaling in SUN-deficient macrophages by adding extracellular Ca^2+^. The results showed that Ca^2+^ entry was significantly higher in *Sun1/2*^*DKO*^ PEMs compared with wildtype PEMs (Fig. 6d). Meanwhile, *Sun1/2*^*DKO*^ PEMs had increased mitochondrial membrane potential (ΔΨm, 2.2-fold) than wildtype PEMs (Fig. 6e), findings consistent with previous studies showing that LPS stimulation of macrophages causes an elevated ΔΨm along with M1 phenotype^34^.

Also, we investigated the potential regulatory effect of SUN1/2 on the metabolic profile of the macrophage. It has been reported that M1-polarized macrophages have increased aerobic glycolysis and lactate production with a high extracellular acidification rate (ECAR); while M2-polarized macrophages preferentially rely on fatty acids oxidation (FAO) and thus have an increased mitochondrial oxygen consumption rate (OCR)^35^. Consistent with the above-described M1-enhancing effect, depletion of SUN1/2 reduced FAO and shifted macrophage metabolism towards aerobic glycolysis, as evidenced by an increased cellular ECAR and reduced OCR (Fig. 6f, Supplementary Fig. 6c).

Together, these observations indicate that decreased levels of SUN protein levels facilitate M1-polarization of macrophages.

### Deficiency of SUN1/2 boosts LPS-induced sepsis and antitumor immunity

**Fig. 7:**
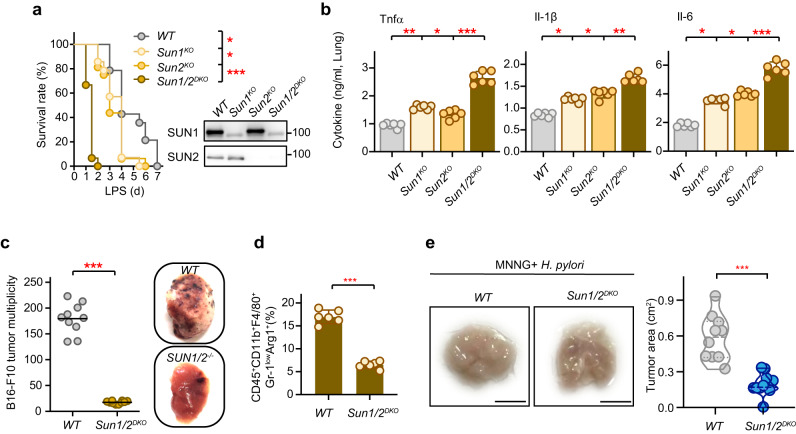
Deletion of SUN1/2 in macrophage boosts inflammation and antitumor immunity in mice. **a** Survival rate of *WT* (*Sun1*^*fl/fl*^*Sun2*^*+/+*^), *Sun1*^*KO*^ (*Sun1*^*fl/fl*^*LysM*^*cre/cre*^), *Sun2*^*KO*^ (*Sun2*^*-/-*^), and *Sun1/2*^*DKO*^ (*Sun1*^*fl/fl*^*LysM*^*cre/cre*^*SUN2*^*-/-*^) mice after LPS challenge (*n* = 20 mice per group). Knockout efficiency of SUN proteins was shown. **b** Cytokine production in the lungs of mice treated with LPS (*n* = 6 murine lungs per group). ELISA analyses of TNFα, IL-1β and IL-6 are shown. **c** Tumor burden of *WT* (*Sun1*^*fl/fl*^*Sun2*^*+/+*^) and *Sun1/2*^*DKO*^ (*Sun1*^*fl/fl*^*LysM*^*cre/cre*^*SUN2*^*-/-*^) mice after B16-F10 cells were injected into their tail veins for ten days (*n* = 10 mice per group). **d** Fraction of M2 macrophages in tumor tissues from *WT* (*Sun1*^*fl/fl*^*Sun2*^*+/+*^) and *Sun1/2*^*DKO*^ (*Sun1*^*fl/fl*^*LysM*^*cre/cre*^*Sun2*^*-/-*^) mice after B16-F10 cells treatment (*n* = 6 mice per group). **e** MNNG/*H. pylori*-induced tumor formation in the stomach of *WT* (*Sun1*^*fl/fl*^*Sun2*^*+/+*^) and *Sun1/2*^*DKO*^ (*Sun1*^*fl/fl*^*LysM*^*cre/cre*^*Sun2*^*-/-*^) mice (*n* = 10 mice per group). Macrophage, MΦ. Kaplan–Meier curve was performed using the logrank test (**a**). Data are presented as mean ± SD (**b**, **d**). Two-sided Tukey post-hoc test was used to compared differences between groups after One-way ANOVA (**b**). Two-sided unpaired student’s *t* test was used to compare difference between two groups (**c**–**e**). *p < 0.05; **p < 0.01; ***p < 0.001, in comparison with control group. See also Supplementary Fig. 7.

To further evaluate in vivo the functional importance of SUN1/2-mediated macrophage polarization, we first examined the response of SUN1/2-deficient mice to LPS-induced septic shock. Considering the functional redundancy of SUN1/2 proteins, we generated mice with SUN1 specifically deleted in macrophage (*Sun1*^*flox/flox*^*LysM*^*cre/cre*^, *Sun1*^*KO*^), SUN2 silenced (*Sun2*^*KO*^), and in combination of these two (*Sun1*^*flox/flox*^*LysM*^*cre/cre*^*Sun2*^*-/-*^, *Sun1/2*^*DKO*^) (Fig. 7a). Flow cytometry analysis showed no significant change in the proportion of CD4^+^ and CD8^+^ splenic thymocytes between wildtype and *Sun1/2*^*DKO*^ mice (Supplementary Fig. 7a). Age- and sex-matched cohorts of mice were then challenged intraperitoneally with LPS and subsequently observed for eight days. The results showed that both *Sun1*^*KO*^ and *Sun2*^*KO*^ mice were more susceptible to dying from LPS-induced septic shock than wildtype mice (Fig. 7a). Moreover, the survival rate of the *Sun1/2*^*DKO*^ mice was further lower than those of either *Sun1*^*KO*^ or *Sun2*^*KO*^ mice (Fig. 7a). Furthermore, adoptive cell transfer assays showed that the median survival time of LPS-treated mice receiving wildtype macrophages was five days, while that of LPS-challenged mice receiving *SUN1/2*^*-/-*^ macrophages was only two days (Supplementary Fig. 7b). Consistent with these observations, LPS-induced production of proinflammatory cytokines (TNFα, IL-1β and IL-6) in the lung tissues from the SUN1/2-deficient mice was markedly elevated compared to wildtype mice (Fig. 7b).

Next, we investigated the potential role of SUN1/2-mediated macrophage polarization in antitumor immunity. To this end, wildtype and *Sun1/2*^*DKO*^ mice were injected with B16-F10 melanoma cells. We noticed a striking decrease in the number of tumors in the *Sun1*^*flox/flox*^*LysM*^*cre/cre*^*Sun2*^*-/-*^ mice compared to wildtype mice (Fig. 7c). Also, the lungs of *Sun1/2*^*DKO*^ mice were substantially lighter than those of littermate controls (Supplementary Fig. 7c), indicating that silencing of SUN1/2 expression markedly reduced tumor load. These observations were further confirmed by a decreased fraction of M2 macrophages (Arg1^+^) in the *Sun1/2*^*DKO*^ mice (Fig. 7d). Moreover, the number of tumors in B16F10-transferred mice receiving *Sun1/2*^*DKO*^ macrophages were also substantially lower than those of mice receiving wildtype macrophages (Supplementary Fig. 7d).

Also, we further assessed the antitumor effect of SUN1/2 depletion in multiple mouse models of primary cancers induced by chemical compound or genetic manipulation. Following a procedure described earlier^36,37^, we treated wildtype and *Sun1/2*^*DKO*^ mice with an alkylating agent methylnitronitrosoguanidine (MNNG) in combination with infection of *Helicobacter pylori* (*H. pylori*) to induce gastric cancer. As expected, the results showed that the average tumor area in *Sun1/2*^*DKO*^ mice was much smaller than those in wildtype mice (Fig. 7e). Since the mammary epithelium-specific transgenic mice expressing the polyomavirus middle T antigen (*PyMT*) can develop spontaneous mammary tumors in multiple mammary glands^38^, we then intercrossed *PyMT* mice with *Sun1*^*flox/flox*^*LysM*^*cre/cre*^*Sun2*^*-/-*^ mice. The amount of time between birth and when tumors were first observed was significantly greater in *Sun1/2*^*DKO*^*PyMT* mice than in wildtype *PyMT* mice (Supplementary Fig. 7e). Consistent with the delayed tumor progression, the average tumor size in the *Sun1/2*^*DKO*^*PyMT* mice was also markedly smaller than in those in control mice (Supplementary Fig. 7f).

Taken together, these results indicate that depletion of SUN1/2 in macrophages exacerbates inflammatory damage in a context of LPS-induced septic shock, while leads to enhanced antitumor immunity in a context of tumorigenesis.

## Discussion

It is generally perceived that the size and stiffness of the nucleus are important for eukaryotic cell fate and function^39,40^. However, the molecular mechanism through which the nuclear size and stiffness are controlled remains elusive. Also, it is unclear how the nuclear size and stiffness change in response to cellular and tissue context, and how these changes, if any, alter the fate and function of a cell. In this work, we found that the nuclear size and stiffness are both acutely reduced during M1 polarization of macrophages. We identified the nuclear envelope proteins SUN1/2 as molecular mediators essential for such nuclear remodeling. Genetic depletion of SUN1/2 decreased the nuclear size and stiffness, promoting M1 polarization of macrophages and thereby boosting inflammation and antitumor immunity (Supplementary Fig. 7h).

The cell nucleus is tightly integrated into the structural network of the cytoplasm through LINC complexes, which mechanically connecting cytoskeleton (i.e., F-actin and microtubule) on one side of the nuclear envelope to nuclear lamina on the other side of nuclear envelope^41^. As key components of LINC complexes, SUN proteins play an essential role in transducing mechanical force between the cytoskeleton and the nucleus^42,43^. Such mechanical connection is thought to be important for a variety of cellular processes. For example, LINC complexes containing SUN2 were found to promote focal adhesion assembly by activating RhoA, a critical regulator for the assembly of F-actin cytoskeleton^44^. A recent study showed that SUN2 played a critical role in mediating mechanical-stress induced nuclear damage and cellular senescence^45^. Here, we further demonstrated a key role for SUN1/2-mediated mechanical changes of the nucleus in macrophage polarization. Our results also implicated the involvement of LINC complexes in this process as the mechanical stress can be transmitted bidirectionally between the nucleus and the cell surface.

Immune cells such as macrophages, may respond to a myriad of biochemical signals, and their function and fate could also be reshaped by mechanical cues. Being present in nearly all types of tissues and sometimes traveling around, macrophages need adapt to tissue environments of varying stiffness, which are often modified under pathological conditions. Although it is increasingly clear that macrophages are mechanosensitive, little is known about how nuclear mechanics modulate their activation. In the current work, we observed that LPS stimulation made the nuclei of macrophages smaller, softer and less coupled with the cytoskeleton, which may facilitate macrophage migrating through narrow stromal environment to exert their patrol functions. We provide evidence that SUN proteins mediate these nuclear changes to drive macrophage polarization. It is possible that during macrophages adapting to a resident tissue, LINC complexes may first transduce the ECM stiffness as a mechanical signal to the nucleus, therefore inducing a series of SUN-mediated nuclear changes to help macrophages either settling down or pushing through. During this process, a temporary “breakdown” of the karyoplasmic ratio appears to be important for the functional reprogramming, i.e., M1 activation of the macrophage. In this regard, note that certain correlations appear to exist between the nuclear sizes and RNA transcription levels^2,46^; and that the activity of DNA polymerase was found to be sensitive for macromolecular crowding^47–49^.

Context-dependent polarization is important for the functional plasticity of macrophages during both physiological and pathological processes. Herein, we demonstrated that SUN1/2-meidated nuclear remodeling controls directional polarization of macrophages. Deficiency of SUN proteins strongly promoted M1 polarization but suppressed M2 polarization of macrophages, leading to enhanced phagocytosis, infiltration, and production of inflammatory cytokines. Genetic deletion of SUN1/2 in macrophages enhanced the antitumor immunity of mice. At molecular level, we revealed that the protein levels of SUN1/2 significantly decreased in macrophages in response to LPS stimulation; and such decreases of SUN1/2 resulted in smaller and softer nuclei of macrophages. Mechanistically, SUN1/2-mediated nuclear remodeling alters chromatin conformation and accessibility to promote gene expressions and metabolic signatures associated with M1 macrophages. At this stage, it remains to be clarified whether SUN1/2-mediated nuclear remodeling is also utilized by other types of cells to control their functional plasticity.

Our study revealed a nuclear envelope-mediated mechanism through which the mechanical nature of the nucleus orchestrates the chromatin conformation and gene expression. In this mechanism, SUN1/2 proteins embedded in the nuclear envelope act as central modulators for the size and stiffness of the nucleus. In keeping with this, SUN1/2 proteins have been found to regulate the shape of the nucleus^50^ and the nucleoli^51^. Notably, we found the crucial role of SUN1/2 proteins in remodeling the nucleus is solely dependent on the transmembrane domain that is embedded in the nuclear envelope. That is to say, all the parts except the transmembrane domain in SUN1/2 are not required for LPS-induced nuclear remodeling and gene expression in the macrophage, emphasizing that SUN1/2 alter chromatin conformation in a mechanical manner, rather than through binding to partner proteins like Lamins and Nesprins. That said, how precisely the transmembrane domain of SUN1/2 regulates the mechanical nature of the nucleus remains unclear.

### Limitation of this study

These results of SUN1/2 mechanoregulation of macrophages are consistent with most previous studies performed in several macrophage cell types and different stiffness ranges^52–54^. Yet we also noticed that our conclusion cannot reconcile with some studies indicating that higher stiffness could shift macrophages from M1 to M2-like phenotype^55,56^. In this regard, we believe that SUN1/2-mediated nuclear mechanics is not a sole factor accounting for LPS-induced biological effects. Rather, it is most likely that combined effects of both nuclear mechanics and cytoskeleton remodeling eventually determine the biological consequences of LPS signaling.

In addition, LPS exposure usually induces reactive oxygen species or reactive nitrogen species to trigger DNA damage response^57,58^ and apoptosis^59,60^. Recently, experimental evidence also shows that LPS stimulation in mouse macrophages could markedly induce cytosolic translocation of PARP1, a key nuclear sensor of DNA damage^61^, which thereby impairs its DNA-binding ability. Moreover, SUN proteins have been previously linked to chromatin dynamics and DNA damage response^62–65^. Knockout of SUN1/2 in MEFs increased apoptosis and DNA damage response, as well as decreased perinuclear heterochromatin^66^. Given that both LPS and SUN proteins have been well documented in genomic instability, it is very likely that LPS might also regulate DNA damages through SUN-mediated nuclear changes, a topic beyond the scope of this manuscript but warrants further investigation.

In summary, our work characterized a phenomenon of nuclear remodeling with a functional role in macrophage polarization and defined SUN1/2 proteins as mechano-regulators required for such nuclear remodeling.

## Methods

### Mice

All animals (six-week old, male: female = 1:1) were randomly assigned to treatment groups in all experiments and performed in accordance with the Institutional Animal Care and Use Committee of Fudan University (approval ID, IDM2022037) and the Institutional Animal Care and Use Committee guidelines of the Animal Core Facility of the Institute of Biochemistry and Cell Biology (Certificate SIBCB-NAF-14-004-S329-023). *Sun1*^*fl/fl*^*Sun2*^*-/-*^ mice (C57BL/6 background) were kindly provided from Prof. Min Han^42,67^. *MMTV-PyMT* transgenic mice and *LysM*^*cre/+*^ mice were obtained from the Jackson laboratory. To generate mice with macrophage SUN1/2 deficiency, we crossed *SUN1*^*fl/fl*^*SUN2*^*-/-*^ mice with *LysM*^*cre/wt*^ mice to obtain *Sun1*^*fl/fl*^*Sun2*^*-/-*^*LysM*^*cre/cre*^ mice (*Sun1/2*^*DKO*^ mice) and their *Sun1*^*fl/fl*^*Sun2*^*+/+*^ littermates (*WT* mice).

### Cells

THP-1 cells and HEK293T cells were purchased from the Cell Resource Center of the Institute of Life Sciences, Chinese Academy of Sciences (Shanghai, China). Human bone marrow-derived macrophages (hBMs, CP-H186) were purchased from Procell (Wuhan, China). B16-F10 murine melanoma cells were kindly provided by Prof. Xiaolong Liu. PEMs were isolated from mice by applying a peritoneal lavage four days after i.p. injection of 1 ml of 3% thioglycollate. Cells (2 × 10^6^) were then obtained from the peritoneal cavities of mice, plated for 2 h in 33 mm cell culture dishes, which were then extensively washed to eliminate cells that did not attach. Bone marrow-derived macrophages (BMDMs) were obtained by flushing tibiae and femurs from mice with ice-cold PBS and passing the suspension through a cell strainer with a 70 μm cut-off. Cells (2 × 10^6^) were plated on 33 mm cell culture dishes in 10 ml complete cultures supplemented with granulocyte-macrophage colony-stimulating factor (GM-CSF) or macrophage colony-stimulating factor (M-CSF) for seven days. THP-1 cells, B16-F10, PEMs and BMDMs were maintained in RPMI1640 medium, whereas HEK293T and MEF cells were in DMEM medium. All cell cultures supplemented with 10% FBS, L-glutamine and penicillin/streptomycin were routinely checked for mycoplasma contamination by using MycoAlert Mycoplasma Detection Kit (LT07-318, Lonza, Rockland, ME). All cell lines were not authenticated after purchase. No commonly misidentified cell lines were used in the study.

### Reagents

IFN-γ (315-05), IL-4 (214-14), M-CSF (315-02) and GM-CSF (315-03) were purchased from Peprotech (Rocky Hill, NJ). LPS (from Escherichia coli, serotype 055:B5, L4005) was purchased from Sigma (St. Louis, MO). The primary antibodies and dilutions used were: Flag (Sigma, F3165, 1:2000), α-tubulin (Sigma, T6199, 1:2000) and β-actin (Sigma, A2228, 1:5000), β-TrCP(Cell Signaling Technology, 4394, 1:1000), Nesprin1 (Santa Cruz, sc-99065, 1:500), Nesprin2 (Santa Cruz, sc-365097, 1:500), LaminA/C (Santa Cruz, sc-7292, 1:1000), ubiquitin (Santa Cruz, sc-8017, 1:1000), CD11b (eBioscience, 17-0112, 1:100), CD86 (eBioscience, 11-0862, 1:100), CD45 (eBioscience, 48-0451, 1:100), F4/80 (eBioscience, 25-4801, 1:100), CD206 (R&D systems, FAB2535P, 1:100) and Arg1 (R&D systems, IC5868P, 1:100) were obtained from. human SUN1 (Abcam, ab103021, 1:1000), mouse SUN1 (Abcam, ab124770, 1:1000) and mouse SUN2 (Abcam, ab124916, 1:1000), and mouse SUN2 (Abcam, ab198981, 1:100, FCS). An antibody specific for human SUN2 (1:1000) was produced by Shanghai Immune Biotech (Shanghai, China).

### Atomic force microscopy

Cells were plated (70% confluence) on 35 mm dishes and stimulated by LPS. After one wash with PBS (room temperature, 10 ml), cells were lysed with 6 ml of hypotonic buffer (10 mM HEPES, 1 mM KCl, 1.5 mM MgCl_2_, 0.5 mM dithiothreitol, and protease inhibitors) and cell bodies were detached using a cell scraper. After incubating for 5 min on ice, samples were homogenized using 30 strokes of a tight-fitting Dounce homogenizer and centrifuged at 700 g for 5 min at 4 °C. Pellets were washed in hyponic buffer and centrifuged again. Then, the nuclear pellet was suspended in buffer S (20 mM HEPES at pH 7.8, 25 mM KCl, 5 mM MgCl_2_, 0.25 M sucrose and 1 mM ATP). For AFM experiments, 10,000 nuclei were plated on a poly-L-lysine-coated coverslip for 30 min at room temperature in 0.5 ml of buffer I (20 mM HEPES at pH 7.8, 25 mM KCl, 5 mM MgCl2 and 1 mM ATP) using the Peak Force Quantitative Nano-Mechanics (PF-QNM) mode on a Bruker Multimode 8 SPM. The silicon nitride DNP-S10 probe was used with a nominal spring constant of 0.35 N/m and a diameter of 20 nm. The PF-QNM mapping AFM images were acquired with a tapping frequency of 0.5–1.0 kHz and the PeakForce tapping amplitude was 300 nm. The scanning speed was about ~1 Hz. To obtain a more precise Young’s modulus (E) value, the deflection sensitivity of the probe was calibrated and the mean value was taken. The spring constant of the probe was calibrated by the thermal noise method in Bruker Nanoscope 9.3 software.

### Electron microscopy

Cells were grown on coverslips before fixation with 2.5% glutaraldehyde for 1.5 h. After fixation, the coverslips were washed with 0.1 M phosphate buffer (pH 7.4) for 3 times (10 min/time). Then the samples were exposed to 1% osmium fixative solution for 1 h at room temperature. Samples were then washed in 0.1 M phosphate buffer (pH 7.4) and dehydrated through an ascending ethanol series. Infiltration was continued using Epon 812 resin in 100% acetone (1:1) and then fresh Epon 812 resin overnight. Thereafter, the samples were embedded in Epon 812 fresh resin and polymerized at 60 °C for 48 h. Ultrathin sections (70 nm) were cut on an ultra-microtome with a diamond knife (Leica EM UC7) and carefully positioned on 100 mesh copper grids (FEI Tecnai G2 Spirit TEM).

### Phagocytosis assay

In vitro phagocytosis assay was performed using Vybrant Phagocytosis Assay Kit (V6694, Thermo) according to the manufacturer’s protocol. Briefly, macrophages were seeded with 5000 cells per well in a 96 well plate (Corning, Tewksbury, MA, USA) in complete RPMI-1640 medium overnight, and then medium was replaced by 100 µl of fluorescent bioparticle suspension containing fluorescent *E. coli* (K-12 strain) bioparticles. Cells were subsequently incubated at 37 °C for 2 h, washed twice with 1 × PBS to remove non-phagocytosed particles, resuspended in 1 × PBS and confocal imagines were captured with a Leica SP8 fluorescent microscope. For each well, at least 100 cells were imaged. Images were than analyzed using Fiji software (ImageJ) to identify single cells and quantify the percentage (%) of phagocytosis-active cells.

### siRNA

Duplexes of siRNA targeting SUN1 and SUN2 and negative controls were synthesized by Genepharma (Shanghai, China). The siRNA sequences are as follows.

For human SUN1, the forward oligo used was 5’-GGAGGGCAGAUAAUUUCAUTT-3’.

For human SUN2, the forward oligo used was 5’-CCCACUGUAUUAUGUAUAUTT-3’.

For the negative control, the forward oligo used was 5’-UUCUCCGAACGUGUCACGUTT-3’.

### Flow cytometry and cell sorting

Cells were put into a 5 ml polystyrene round-bottom tube and stained for 30 min at 4 °C with indicated antibodies. Intracellular staining was performed after 10 min fixation (2% formaldehyde PBS) at room temperature and 5 min permeabilization in IC staining buffer (0.1% saponin, 0.1% bovine serum albumin Hank’s balanced salt solution) at 4 °C. Cell fluorescence was determined using a two-laser FACS Calibur (BD Biosciences, Mississauga, ON, Canada) flow cytometer, and data were analyzed with FlowJo software (TreeStar, Olten, Switzerland). M2 macrophages and TAM cells were sorted using a FACS Aria II cell sorter (BD Biosciences), and the cell purity was consistently greater than 90%.

### Transfection and reporter assay

HEK 293 T cells were transfected with plasmids encoding NF-κB luciferase or pRL-TK Renilla luciferase and different expression or control vectors. Lipofectamine 2000 and lipofectamine LTX with PLUS reagent (Invitrogen, Carlsbad, CA) were used. The luciferase activity was determined by using a dual luciferase assay kit (Promega, Madison, WI) with a Luminoskan Ascent luminometer (Thermo Scientific, Waltham, MA).

### RNA isolation and quantitative real-time PCR (QPCR)

Cells were plated on two kind of cell plates with collegen-coated polyacrylamide hydrogels of varying stiffness, 1 and 50 kPa (Matrigen, Brea, CA, USA) for attachment. Total RNA was then extracted with Trizol reagent (Invitrogen). For nuclear RNA, the nuclei fractions were isolated as described for the AFM imaging. Subsequently, the nuclear RNA was extracted using a spin-based Rneasy Micro Kit (Qiagen) after incubation for 60 min at 37 °C in the 3D Matrigel. All RNA purity and quantity was determined using a NanoDrop spectrophotometer (ND-1000; NanoDrop Technologies). the real-time PCR was performed using an Applied Biosystems Step Two Real-Time PCR System (Applied Biosystems) and using the comparative Ct quantization method and compared with internal control. GAPDH was used as an internal control. Three biological replicates were used for analysis, and all reactions were run in triplicates. The primers used were as follows:

m*Sun1*, 5′-CACTGGCTACACTTACGCACT-3′(F)

5′-CCACTGCTGTACGAAGCTGTT-3′(R)

m*Sun2*, 5′-ACCTACAGCCGTTACCTTAGAG-3′(F)

5′-TCGAAGCCAAAGATGGCGAAG-3′(R)

m*Nos2*,5′-GTTCTCAGCCCAACAATACAAGA-3′(F),

5′-GTGGACGGGTCGATGTCAC-3′(R)

m*Tnfa*:5’-CAGGCGGTGCCTATGTCTC-3’ (F),

5’-CGATCACCCCGAAGTTCAGTAG-3’ (R);

m*Il1b*:5’-GAAATGCCACCTTTTGACAGTG-3’(F),

5’-TGGATGCTCTCATCAGGACAG-3’ (R);

m*Il1b* (-990 ~ -868): 5’-GCCAAGAGACTTGGTCTCCCC-3’ (F),

5’-ACGAGGCATCTGCCTGTTCA-3’ (R);

m*Il1b* (-881 ~ -741): 5’-GGCAGATGCCTCGTTCACCA-3’ (F),

5’-CCACTCCTGCTTTCCTGCCC-3’ (R);

m*Il1b* (-759 ~ -665): 5’-GGCAGGAAAGCAGGAGTGGG-3’ (F),

5’-CCAGGTCTCCCCTCCGGAAA-3’ (R);

m*Il1b* (-585 ~ -501): 5’-AGGCTTGCTTCCAGAGTTCCC-3’ (F),

5’-CTTGTGTGGGTCAGGGCACA-3’ (R);

m*Il1b* (-495 ~ -348): 5’-GCGTGTCTCTCCAGAAGCCC-3’ (F),

5’-GGCACGTAGATGCACACCCA-3’ (R);

m*Il1b* (-268 ~ -179): 5’-GCACAATTGTCCAGGGGGAA-3’ (F),

5’-CCTGGAAGTCAAGGGGTGGC-3’ (R);

m*Il1b* (-114 ~ 16): 5’-CCCTCCCCCACCCTTCAGTT-3’ (F),

5’-CCACTGCAGGGTTTGTTGTCC-3’ (R);

m*Il1b* (40 ~ 169): 5’-GGGATCCTCTCCAGCCAAGC-3’ (F),

5’-ACAGAGAGAGAGACAGACAGAGACA-3’ (R);

m*Il1b* (199 ~ 311): 5’-TCTGTCTCTGTCTCTCTCTGTCTGT-3’ (F),

5’-GCCCAAAGTCCATCAGTGGGG-3’ (R);

m*Il1b* (371 ~ 454): 5’-ACTGTCTGTATAGCCGCTGACAT-3’ (F),

5’-GCAACAGCAGAGCCAAACCC-3’ (R);

m*Il1b* (601 ~ 707): 5’-GACCCCTGTGAAAGGGCCAC-3’ (F),

5’-GGGCAGGCATGCTAAACTGGT-3’ (R);

m*Il1b* (657 ~ 767): 5’-ACGGCTCCTCCGTTCCTTCA-3’ (F),

5’-CATCCAGCGTTAGCTCCCCG-3’ (R);

m*Il1b* (748 ~ 882): 5’-CGGGGAGCTAACGCTGGATG-3’ (F),

5’-TGTGACCACTCTCCAGTACCCA-3’ (R);

m*Il1b* (895 ~ 983): 5’-TGCTTTCAGGAATGGAGGGCT-3’ (F),

5’-AGGTTGCTTGAACTCTGATAGCCA-3’ (R);

m*Il6*: 5’-TCTATACCACTTCACAAGTCGGA-3’ (F),

5’-GAATTGCCATTGCACAACTCTTT-3’ (R);

m*Il6*(-996 ~ -895): 5’-GCTAAGATACAATGAGGTCCTTCTTCG-3’ (F),

5’-GGCTTGAAGGTCTGTTGCAGT-3’ (R);

m*Il6* (-889 ~ -810): 5’-GCATGACCTGGAAATGTTTTGGGG-3’ (F),

5’-AGTCTCTGTGAGAGTTGCCCTT-3’ (R);

m*Il6* (-862 ~ -769): 5’-CCTGGCAGCAGTGGGATCAG-3’ (F),

5’-TCCCCAGTGGTCTCTTGGCT-3’ (R);

m*Il6* (-788 ~ -667): 5’-AGCCAAGAGACCACTGGGGA-3’ (F),

5’-TCCAGGAGTTGCCAGGTGGG-3’ (R);

m*Il6* (-685 ~ -551): 5’-CCACCTGGCAACTCCTGGAA-3’ (F),

5’-CCAGCACCCAACCTGGACAA-3’ (R);

m*Il6* (-570 ~ -425): 5’-TTGTCCAGGTTGGGTGCTGG-3’ (F),

5’-ACACACACACACACACACACACA -3’ (R);

m*Il6* (-378 ~ -278): 5’-GCGCGTGCCTGCGTTTAAATA-3’ (F),

5’-AGTCTCTGTGAGAGTTGCCCTT-3’ (R);

m*Il6* (-233 ~ -133): 5’-AGGGCTAGCCTCAAGGATGACT-3’ (F),

5’-GAGTGGGTGGGGCTGATTGG-3’ (R);

m*Il6* (-108 ~ 23): 5’-CACCCCCACCCTCCAACAAA-3’ (F),

5’-CTTGGTGGGCTCCAGAGCAG-3’ (R);

m*Il6* (3 ~ 108): 5’-TCTGCTCTGGAGCCCACCAA-3’ (F),

5’-CAATAGCTCCGCCAGAGGGC-3’ (R);

m*Il6* (89 ~ 178): 5’-GCCCTCTGGCGGAGCTATTG-3’ (F),

5’-GCTGCTAGCTGATGGCTGCT-3’ (R);

m*Il6* (-378 ~ -278): 5’-GCGCGTGCCTGCGTTTAAATA-3’ (F),

5’-AGTCTCTGTGAGAGTTGCCCTT-3’ (R);

m*Il6* (-233 ~ -133): 5’-AGGGCTAGCCTCAAGGATGACT-3’ (F),

5’-GAGTGGGTGGGGCTGATTGG-3’ (R);

m*Il6* (180 ~ 288): 5’-GGCGCCCAACTGTGCTATCT-3’ (F),

5’-AAGGCCGTGGTTGTCACCAG-3’ (R);

m*Il6* (273 ~ 373): 5’-TGACAACCACGGCCTTCCCT-3’ (F),

5’-GCCTCCGACTTGTGAAGTGGT-3’ (R);

m*Il6* (435 ~ 515): 5’-CTGATGAAGACCCAGTGTGGGC-3’ (F),

5’-AAGGGCCCTAGATCCCAGCA-3’ (R);

m*Il6* (593 ~ 672): 5’-AAGGGGTTCCTTTCCTGTCTGG-3’ (F),

5’-TGGAACAGAGAATGGCCCACTG-3’ (R);

m*Il6* (820 ~ 901): 5’-GGATGCTCTAGGGTCAGCCCA-3’ (F),

5’-TGTGTGTGTGTGTGTGTGTGTGT-3’ (R);

m*Il6* (887 ~ 974): 5’-ACACACACACACACACACACACA-3’ (F),

5’-ATCTTCCTGCGTGTGCCTCC-3’ (R);

*mArg1*,5′-CTCCAAGCCAAAGTCCTTAGAG-3′(F),

5′-AGGAGCTGTCATTAGGGACATC-3′(R);

*mRetnla*, 5′-CTGGGTTCTCCACCTCTTCA-3′(F),

5′-TGCTGGGATGACTGCTACTG-3′(R);

*mMrc1*, 5′-CTCTGTTCAGCTATTGGACGC-3′(F),

5′-CGGAATTTCTGGGATTCAGCTTC-3′(R);

*mCTGF*, 5′-GGCCTCTTCTGCGATTTCG-3′(F),

5′-GCAGCTTGACCCTTCTCGG-3′(R);

*mCyr61*, 5′-GATGACCTCCTCGGACTCGAT-3′(F),

5′-CGTGCAGAGGGTTGAAAAGAA-3′(R);

*mAxin2*, 5′-ATGAGTAGCGCCGTGTTAGTG-3′(F),

5′-GGGCATAGGTTTGGTGGACT-3′(R);

*mMafb*,5′-GCAACGGTAGTGTGGAGGAC-3′(F),

5′-TTCAGGCGGATCACCTCGT-3′(R);

m*GAPDH*: 5’-TTGTCATGGGAGTGAACGAGA-3’ (F),

5’-CAGGCAGTTGGTGGTACAGG-3’(R) (F, forward; R, reverse).

### RNA-sequencing

HGC-27 cells in 6-well plates were treated with GLUP peptide at 10 μg/ml for 48 h. The solvent served as negative control. Total RNA was extracted from three biological replicates. RNA quality was assessed using a 2100 Expert Bioanalyzer (Agilent) and sent for library preparation and sequencing using the Illumina Hiseq2000 platform of Majorbio Biotech (Shanghai, China). The data were analyzed on the free online Majorbio I-Sanger Cloud Platform (www.i-sanger.com).

### FAIRE assay

The samples were sonicated using a Q800R3 Sonicator (Active Motif, USA), which yielded DNA fragments with an average size of 250–500 bp. qPCR was conducted as described earlier using 40 ng of DNA recovered from crosslinked cells and non-crosslinked reference cells. All samples were analyzed in duplicate, and the data were calculated as percent input. Primer sequences and PCR conditions are listed in “RNA Isolation and Quantitative Real-time PCR (QPCR)” subsection.

### Chromosome conformation capture (3 C) assay

Cells were treated with 2% formaldehyde for 5 min and then stopped by the addition of glycine (0.125 M) for 5 min at room temperature. Nuclei were prepared by adding 30 ml of lysis buffer (10 mM Tris/HCl, 10 mM NaCl, 0.2% NP40 pH 8.0, 0.1 mM PMSF, 1:500 protease inhibitor) to the cell pellet. Nuclei were then incubated at 4 °C for 90 min, centrifuged at 2500 rpm for 15 min and re-suspended in 200–300 μl of 1 × NEB buffer 3 (NEB, UK) containing 0.3% SDS to ensure lysis of nuclear membrane. Nuclei were counted, incubated at 37 °C on a shaker and SDS was sequestered by adding Triton X to a final concentration of 1.8% and incubated again at 37 °C. Aliquots of 1 million nuclei were digested separately in 0.5 ml tubes by adding 600 units of highly concentrated Bgl II in a total volume of 70 μl and incubated over- night at 37 °C in a shaker. Bgl II was inactivated by adding SDS to a final concentration of 1.6% and incubated for 20 min at 65 °C. Digested DNA was diluted to 2.5 ng/ml with 1 × ligation buffer (30 mM Tris, 10 mM MgCl_2_, 10 mM DTT and 1 mM ATP, pH 8) containing 1% Triton-X and incubated for 1 h at 37 °C while gently shaking. The temperature was lowered to 16 °C in a water bath and 30 Weiss units of T4 DNA ligase were added for 4 h at 16 °C. To reverse the crosslinking, proteinase K was added to a final concentration of 100 μg/ml and incubated at 65 °C overnight. Further purification involved RNase A treatment (0.5 μg/ml, 1 h 37 °C), followed by phenol chloroform extraction and ethanol precipitation. DNA was then desalted and concentrated using centrifugal ultrafiltration.

Primers for 3C were designed according with the parameters that optimal length between 19 and 25 bp long, Tm of 60 ± 2 °C, and GC content limited to 43–51%. We designed two primers for each Bgl II restriction site, one on each side in position of 45–140 bp from the Bgl II restriction site. Primer sequences were as follows:

a1: 5′-CGCCATGAAAATTGGATGT-3′; a2: 5′-CCCAGAAGCCAATGAAGAAC-3′; a3: 5′-AAGTAGGCTGCAGAGCAATCA-3′; a4: 5′-TGGCCCATAAAACCTCTGG-3′; b1: 5′-TCCAGGAGAATGACCTGAGC-3′; b2: 5′-ACCGCACAAACAGTAAATGCT-3′; b3: 5′-CACCATGTGGACAGGAGATG-3′; b4: 5′-ATGTGTCAATCCTGCCCCTA-3′

### ELISA

Serum and cell culture supernatants were collected and assayed for cytokines. Cytokine production was measured by performing enzyme-linked immunosorbent assays (ELISA) of human (eBioscience) or mouse (BD Bioscience) TNF-α, IL-1β and IL-6 according to the manufacturer’s protocol.

### Immunoblotting

For immunoprecipitation experiments, whole cell extracts were prepared after transfection or stimulation, and incubated overnight with indicated antibodies together with Protein A/G beads (Santa Cruz Biotechnology). Beads were then washed three times with lysis buffer, and immunoprecipitates were eluted with SDS loading buffer and resolved in SDS-PAGE gels. The proteins were transferred to a PVDF membrane (Bio-Rad) and further incubated with the indicated antibodies.

For the immunoblotting analysis of actin (de)polymerization, detergent-soluble and detergent-insoluble cell extracts were prepared as follows. Cells were lysed with a buffer containing 50 mM Pipes/KOH (pH 6.9), 50 mM NaCl, 5 mM MgCl_2_, 5 mM EGTA, 5% glycerol, 0.1% Triton X-100, 0.1% Tween 20, 0.1% (v/v) 2-mercaptoethanol, 1 mM ATP and protease inhibitors. After incubation for 10 min at 37 °C, samples were centrifuged at 100,000 g for 60 min at room temperature. Supernatants containing detergent-soluble cell proteins were harvested and put on ice. Pellets of detergent-insoluble proteins were resuspended in the same volume as their supernatants with ice-cold lysis buffer.

### Immunofluorescence

Macrophages (2 × 10^5^) were seeded onto coverslips in six-well dishes and grown for 8 h before stimulation with 1 μg/ml LPS. Cells were fixed with 4% paraformaldehyde, followed by ice-cold methanol. Cells were stained with the indicated antibody, and then were counterstained for nucleic acids with DAPI. Images were captured with a Zeiss LSM 710 laser-scanning confocal microscope or NIKON N-SIM microscopy system. The fluorescence intensity within a cell was evaluated with Fiji software (ImageJ). For each group, three images were acquired, and at least 25 cells were analyzed. The fold change of fluorescence intensity was calculated as ratios of fluorescence intensity values of LPS-stimulated group to fluorescence intensity values of unstimulated controls.

### In vitro cell infiltration

Cell infiltration was mimicked in vitro by using a Transwell chamber. In brief, macrophages (1 × 10^5^) were harvested and placed in the upper chamber coated with 1–2 mg/ml Matrigel (reconstituted basement membrane; BD Biosciences). Twenty-four hours later, the cells in the upper chamber were removed with a cotton swab. The remaining cells on the membrane were fixed for 10 min in methanol, stained with 1% crystal violet solution and washed with PBS. For quantification, infiltrated cells were soaked in 200 μl of DMSO and subjected to optical density measurement at 450 nm using DMSO as a blank control.

### FISH

FISH was performed according to the protocol of FISH assay kit (Abnova, Taiwan). Briefly, cells were placed on the slides, which were sequentially treated with methanol:acetic acid, 2× standard saline citrate, hot citric acid, and pepsin. Centromere-specific α-satellite (CEN) probes for chromosomes 1 (green) and chromosome 5 (red) were added to each slide, the DNA and probe were co-denatured and hybridized, and the slides were washed and counterstained. Cells were then viewed with a Leica SP8 fluorescent microscope.

### Seahorse assay

To measure ECAR and OCR in a real-time manner, cells were isolated from mice, seeded at 1 × 10^5^ cells/well in eight-well miniplate format and allowed to adhere overnight. One hour prior to reading, cells were washed twice, and then cultured in Seahorse XF base medium (Agilent Technologies) supplemented with 1 mM pyruvate, 2 mM L-glutamine and 10 mM glucose in an incubator without CO_2_. ECAR and OCR were measured under basal conditions prior to sequential treatment of cells with electron transport chain inhibitors 1 μM oligomycin, 1.5 μM FCCP-cyanide p-tribluromethoxyphenyl-hydrazone, and 1 μM antimycin A and rotenone (Seahorse XF Cell Mito Stress Test kit, Agilent, #103015-100). Data represent mean ± SD of triplicate wells from at least three individual mice.

### Animal model

Chow-fed, six week-old male C57BL/6 mice were housed under a reverse light-dark cycle. The mice were injected via the tail vein with 5 × 10^5^ B16-F10 melanoma cells. Lung and plasma were collected ten days after administration of the cells. For LPS challenge, LPS (5 mg/kg) was administered intraperitoneally. Survival of the mice was monitored twice a day for 7 days. Mice used for time-point studies were sacrificed and their serum and lungs were collected.

For MNNG/*H.pylori*-induced gastric cancer (GC) Model, C57BL6 mice were housed in an air-conditioned biohazard room designed for infectious animals, with a 12 h Light: 12 h Dark cycle. The GC mice model was established following a previously established protocol^37^. For each cycle, drinking water containing MNNG (100 mg/ml) was served for the mice for 14 consecutive days, and then normal drinking water was served for next 14 days. Moreover, mice were intragastrically administrated with *H. pylori* SS1 (1 × 10^7^ CFU/ml) for 14 days during each cycle. Three cycles of treatment were carried out to establish GC mice model. After 180 days of treatment, mice were scarified for subsequent analysis. The tumor size per stomach were counted (*n* = 10 per group).

### Murine sepsis model

Male C57/BL6 mice (8 weeks old) were purchased from SLAC Laboratory Animal (Shanghai, China) in this study. Sepsis was induced by intraperitoneal (*i.p*.) administration of bacterial LPS (Sigma) from the *Escherichia coli* strain 055: B5 (20 mg/kg) dissolved in sterile saline. This procedure has been previously used to generate LPS-induced murine sepsis models^68^.

### B16-F10 melanoma lung metastasis model

B16-F10 cells (3 × 10^5^ cells in 0.1 ml of saline) were injected into mouse tail vein. Subsequent adoptive transfer was performed by injecting PEMs (5 × 10^6^ cells in 0.1 mL of saline) into the mouse tail vein at day 1, 5 and 10. Two weeks after B16-F10 cell injection, mice were euthanized, and both left and right lung lobes were dissected under a stereomicroscope to count the number of metastatic nodules.

### *PyMT* tumor model

We crossed *Sun1*^*fl/fl*^*;Sun2*^*-/-*^*;LysM*^*cre/cre*^ mice with *MMTV-PyMT* mice (Jackson, 002374) after at least three generations of backcrossing to obtain *MMTV-PyMT;Sun1*^*fl/fl*^*;Sun2*^*-/-*^*;LysM*^*cre/cre*^ mice (*Sun1/2*^*DKO*^*PyVT*). *MMTV-PyMT* (*PyMT*) mice and *Sun1/2*^*DKO*^*PyVT* female mice were identified. Mice were palpated twice weekly for the appearance of mammary tumors. The tumor free time was calculated as the age at which the first tumor was palpated. Palpable mammary tumors were measured every 5 days, and mice were sacrificed at the fourth month after birth. Tumors were harvested, weighed and stored for further analysis. Tumor volumes were calculated as Volume (mm^3^) =   0.5 × length × width^2^.

### Statistical and reproducibility

In general, at least two independent experiments were performed with similar results. No statistical methods were used to predetermine sample sizes and sample sizes were chosen empirically. No data were excluded from the analyses. For in vivo experiments, all mice were randomly allocated into different experimental groups. For in vitro studies, no randomization was performed. The investigators were not blinded to allocation during experiments and outcome assessment. Data are shown as mean ± S.D. Continuous data were compared using Student’s *t* tests (when comparing two variables) or one-way ANOVA (when comparing three or more variables). To determine correlations, the Spearman rank correlation was used for continuous variables. Survival curves were calculated according to the Kaplan–Meier method; survival analysis was performed using the logrank test. A value of *p* < 0.05 was considered to indicate a significant difference.

### Reporting summary

Further information on research design is available in the Nature Portfolio Reporting Summary linked to this article.

### Supplementary information

Supplementary Information

Description of Additional Supplementary Files

Supplementary Movie 1

Supplementary Movie 2

Reporting Summary

### Source data

Source Data

## Data Availability

The sequencing data generated in this study have been deposited in the Gene Expression Omnibus (GEO) under accession code GSE85022, GSE243684. All other data are available in the article and its Supplementary files or from the corresponding author upon request. Source data are provided with this paper.
