# Supplementary material for: SUN1/2 controls macrophage polarization via modulating nuclear size and stiffness
Source: Nat Commun. 2023 Oct 12;14:6416. doi: 10.1038/s41467-023-42187-5 (PMC10570371; doi:10.1038/s41467-023-42187-5)
Supplement: Supplementary file 1 — Supplementary Information [file 41467_2023_42187_MOESM1_ESM.pdf]

Supplemental Information

Supplementary figures (1~7) and legends

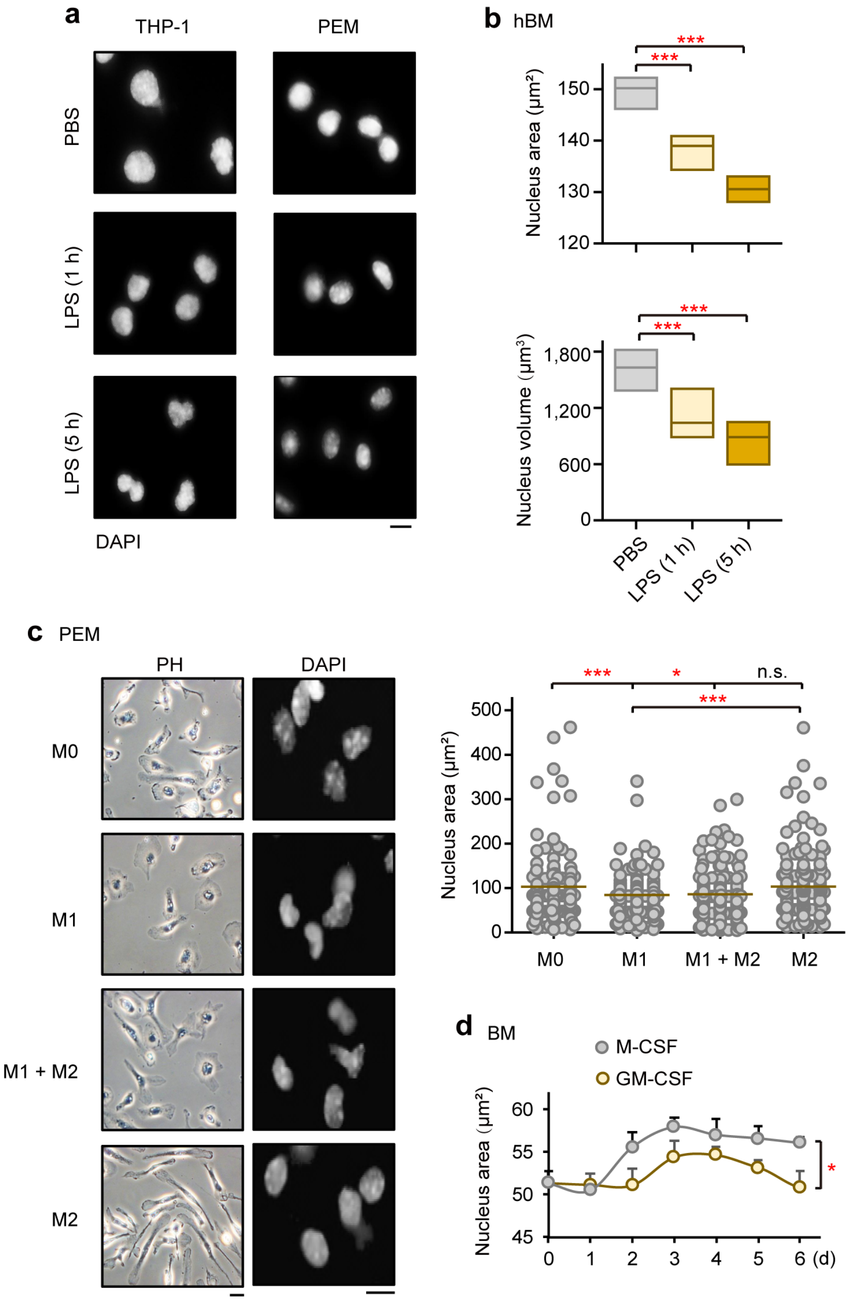

Supplementary Fig. 1 LPS treatment decreases the average size of nuclei in macrophages. **a** DAPI staining for the indicated macrophages. Macrophages were fixed and stained with DAPI after LPS stimulation. Scale bar, 5 μm. **b** The nucleus size of the human bone marrow-derived macrophages (hBM) upon LPS stimulation (n=50 cells per group). The nucleus area (upper) and volume (lower) were quantified, respectively. Box plots show the median, minimum and maximum values. LPS, 100 ng/ml. **c** Fluorescence microscopy (DAPI staining) and phase-contrast (PH) showing the size of the nuclei of PEMs before or after M1 or M2 polarization (n=500 cells per group). The horizonal line corresponds to the mean value. Scale

bar, 5 µm. **d** Average size of nuclei of bone marrow monocytes subjected to GM-CSF- or M-CSF-stimulated differentiation for six days ((n=500 cells per group). Two-sided Tukey post-hoc test was used to compared differences between groups after One-way ANOVA (b, c). Data were presented as means+SD (d). Unpaired student's *t*- test was used to compared difference between two group (d). *, $p<0.05$; **, $p<0.01$; ***, $p<0.001$, n.s., no significance ($p>0.05$) in comparison with control group. Related to Fig. 1

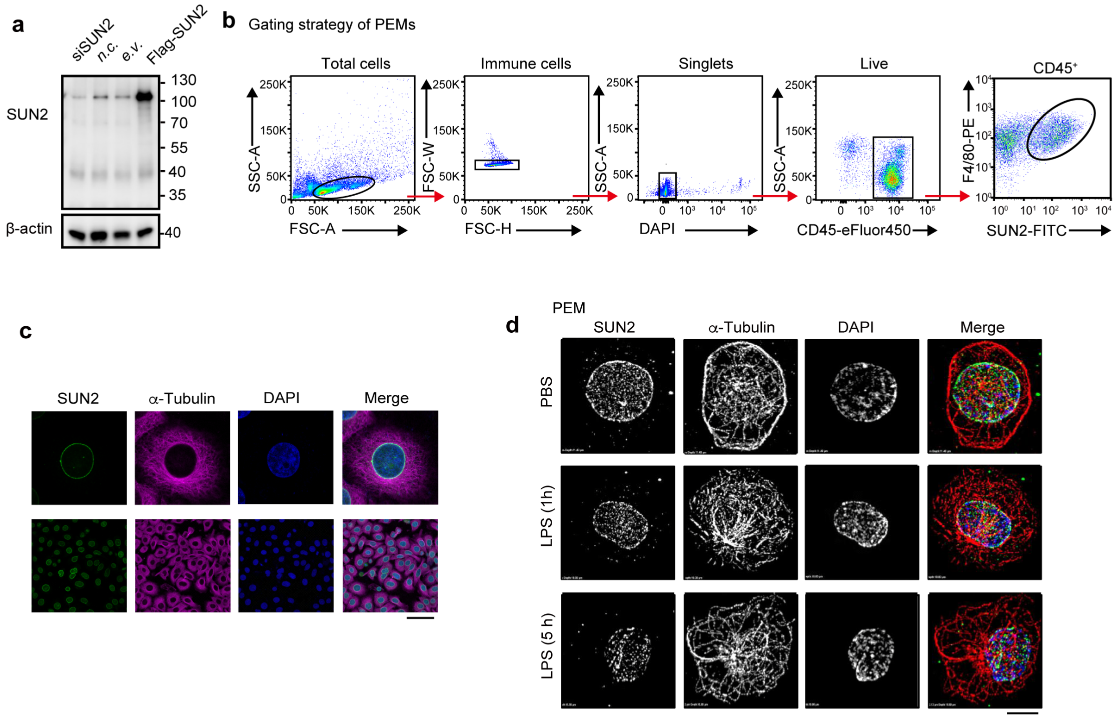

Supplementary Fig. 2 LPS treatment decreases SUN2 protein level. a Immunoblot for SUN2 in cells transfected with Flag-SUN2 and SUN2-specific siRNA. **b** Gating strategy of PEMs. **c** Representative immunofluorescent image of SUN2 (green) and α-tubulin (purple) in Hela cells. Scale bar, 10 μm. **c** Expression levels of the indicated proteins in THP-1-derived macrophages. **d** Immunofluorescent staining of SUN2 (green) and α-tubulin (red) in LPS-stimulated PEM. Scale bar, 10 μm. Representative of 2 independent experiments (a-d). Related to Fig. 2

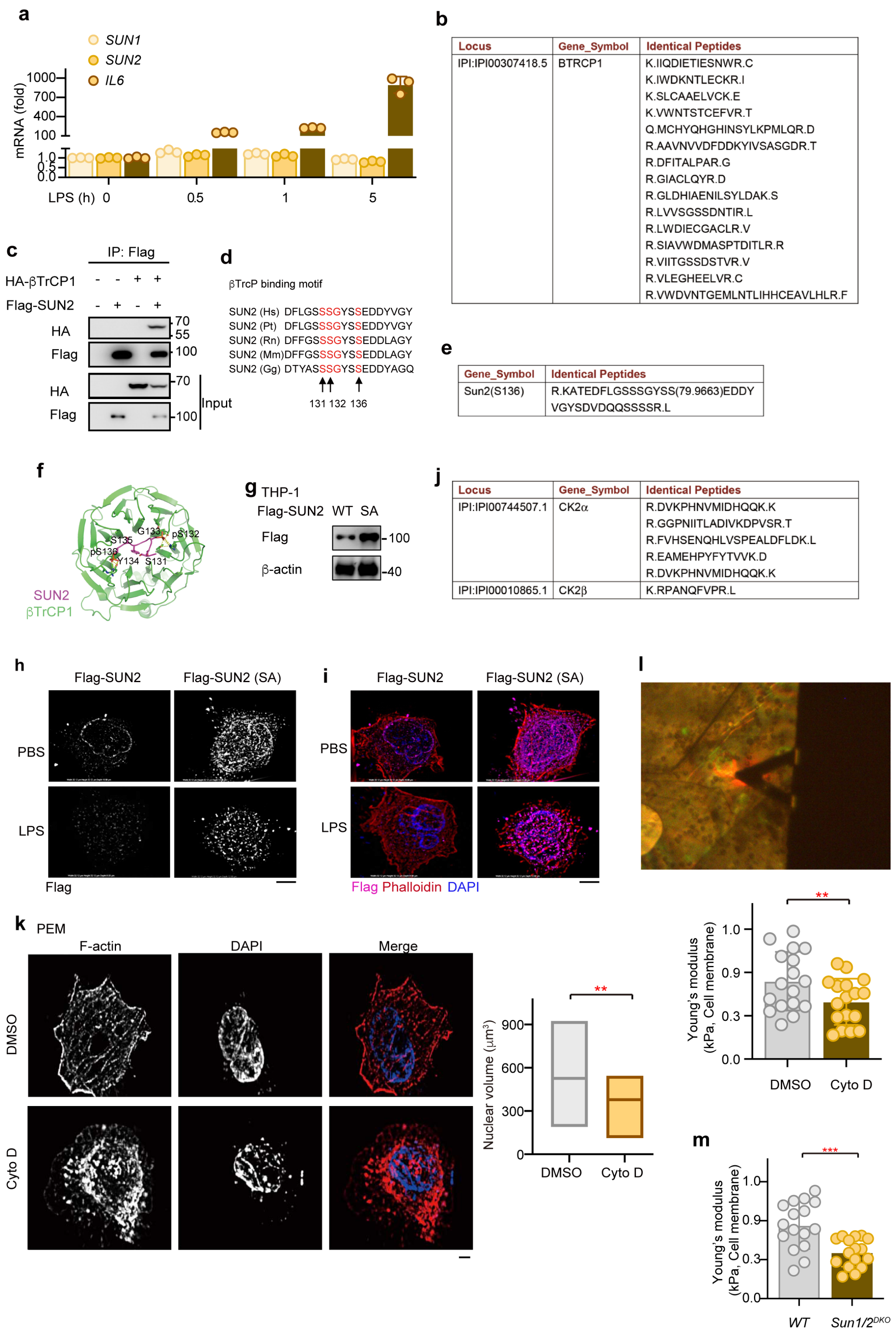

Supplementary Fig. 3 The E3 ubiquitin ligase βTrCP promotes SUN2 degradation in manner dependent on CK2 phosphorylation of SUN2. a mRNA levels of Sun1, Sun2 and Il-6 in THP-1-derived macrophages after treatment with LPS (n=3 biological replications per group). **b** A list of βTrCP peptides from the mass spectrometry (MS) of SUN2

immunoprecipitates. **c** Interaction of Flag-Sun2 with HA-βTrCP1. **d** Sequence alignment of human, chimpanzee, rat, mouse and chicken SUN2 βTrCP-binding-motif regions. **e** Mass spectrometry analysis of SUN2 phosphorylation. **f** Modeled structure of SUN2 bound with βTrCP. **g** Immunoblotting showing the expression of Flag-SUN2 and its SA mutant. **h** Immunofluorescent staining for Flag-SUN2 and its SA mutant in the THP-1-derived macrophages upon LPS (1 μg/ml) stimulation for 5 h. Scale bar, 5 μm. **i** Co-staining of Flag, Phalloidin and DAPI in THP-1-derived macrophages after LPS stimulation. Scale bar, 5 μm. **j** A list of CK2 peptides from the Mass spectrometry analysis of SUN2 immunoprecipitates. **k** Confocal images of PEMs after treatment with DMSO or cytochalasin D (Cyto D, 2 μM) for 1 h. The cells were stained with anti-F-actin (red) and counterstained with DAPI (blue). Box plots showing the median, minimum and maximum values. Scale bars, 5 μm. **l** The Young's modulus of PEMs treated with DMSO and Cyto D (n=16 cells per group). n=16. **m** The Young's modulus of *WT* and *Sun1/2*^{DKO} PEMs (n=16 cells per group). Data were presented as means±SD (a,l,m). Representative of 2 independent experiments (c,g). Two-sided unpaired student's *t*- test was used to compare difference between two groups (k,l,m). **, p<0.001; ***, p<0.001. Related to Fig. 3

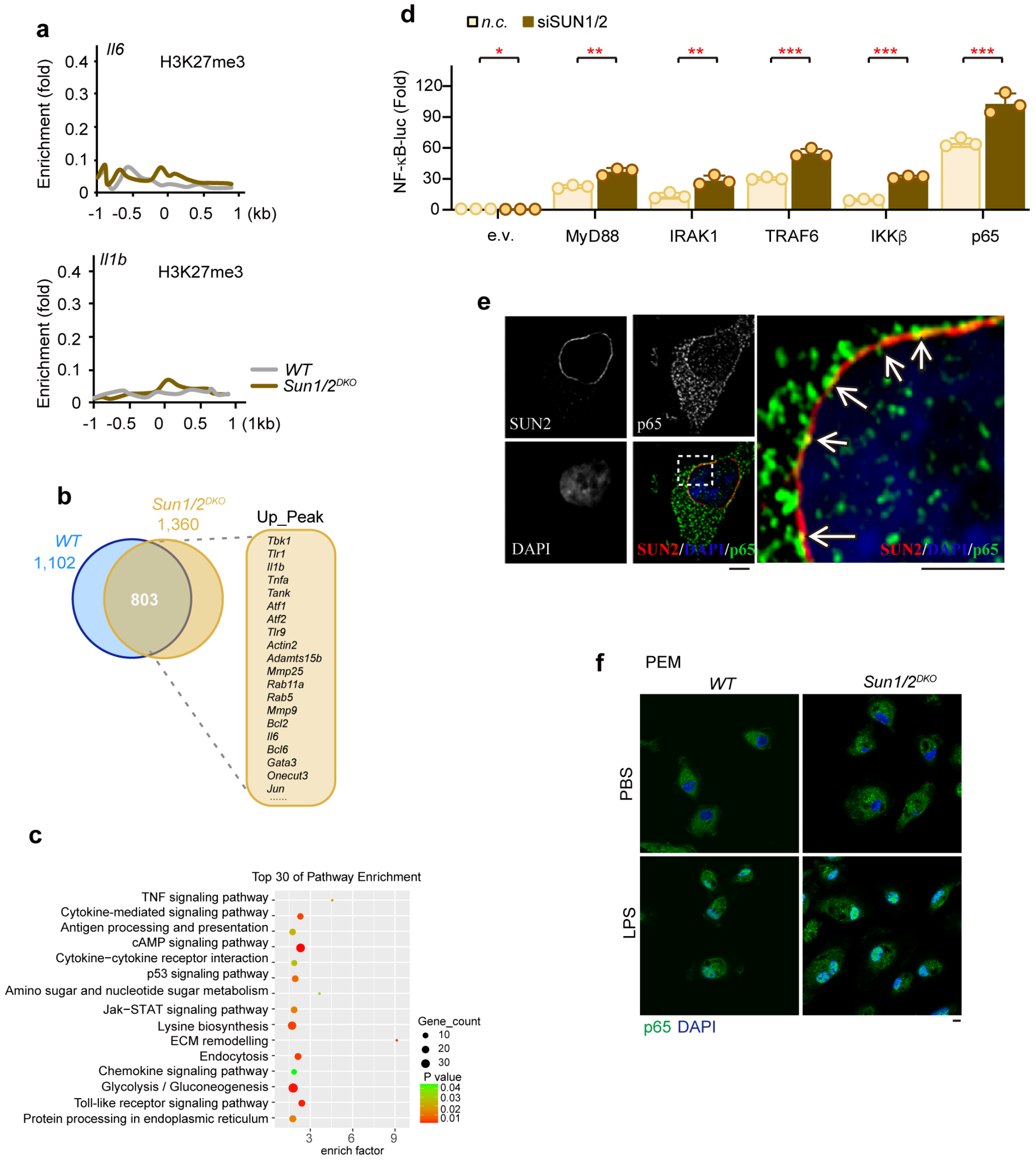

Supplementary Fig. 4 SUN depletion promotes chromatin openness. a ChIP assay to analyze the enrichment of H3K27me3 on *Il6* and *Il1b* loci in wildtype and *Sun1/2*^{DKO} PEM. **b** Venn diagram illustrating the overlapping peaks of ChIP-seq data in wildtype and *Sun1/2*^{DKO} PEMs. The top 20 genes were shown. **c** KEGG analysis of H3K4me3 peaks showing the top 15 enriched pathways. **d** Luciferase activity detected in SUN1/2-knockdown HEK293T cells co-transfected with a NF-κB luciferase reporter, plus empty vector or MyD88, IRAK1, TRAF6, IKKβ or p65 (n=3 biological replications per group). **e** Co-staining of SUN2 and p65 in the THP-1-derived macrophages. **f** Nuclear translocation of p65 in wildtype or SUN1/2-knockout PEMs treated with or without LPS. Data were presented as means±SD (d). Representative of 2 independent experiments (e, f). Related to Fig. 4

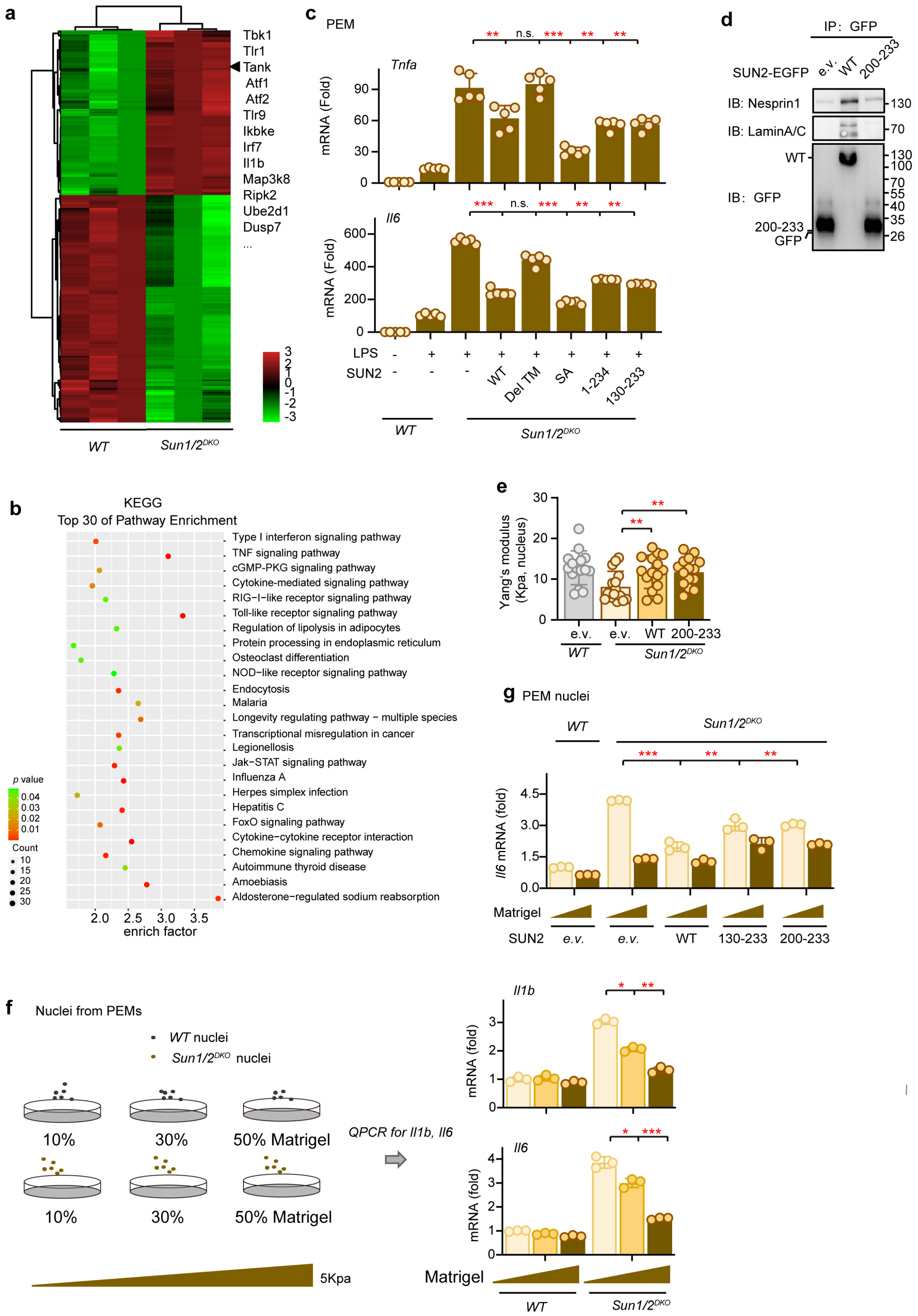

Supplementary Fig. 5 The regulatory effect of SUN1/2 on gene expression and cellular stiffness of macrophages. a Expression of genes encoding products in PEM (*WT* and *Sun1/2*^{DKO}) after treatment with LPS, showing genes changed in SUN1/2-deficent PEM after LPS stimulation relative to their expression in *WT* PEM (change in expression of >2-fold; p < 0.05). **b** KEGG analysis showing the enriched pathways. **c** Rescue of the SUN-mediated

suppression of the LPS-induced transcription of *Tnfα* and *Il6* in *Sun1/2*$^{-/-}$ PEMs by transfection of wildtype SUN2 and its mutants (n=5 biological replications per group). Wild type PEMs were presented for comparison. "Del TM" represents a SUN2 mutant in which the transmembrane domain was deleted. Note that the 130-233 SUN mutant were presumably disabled for binding to both Nesprins and Lamins. **d** Co-IP of LaminA/C and Nesprin1 with SUN2 and its truncation in PEMs. **e** The nuclear Young's modulus of the indicated PEMs after transfection with SUN2 and its truncation (n=16 nuclei per group). **f** Analysis of mechanosensitivity of cytokine transcription in separated PEMs nuclei by using different concentration of matrigel (n=3 biological replications per group). **g** Analysis of mechanosensitivity of *Il6* transcription in the indicated PEMs nuclei by using different concentration of matrigel (n=3 biological replications per group). Data were presented as means±SD (c, e-g). Two-sided Tukey post-hoc test was used to compared differences between groups after One-way ANOVA (c, e-f). *, $p<0.05$; **, $p<0.01$; ***, $p<0.001$, n.s., no significance ($p>0.05$) in comparison with control group. Related to Fig. 5

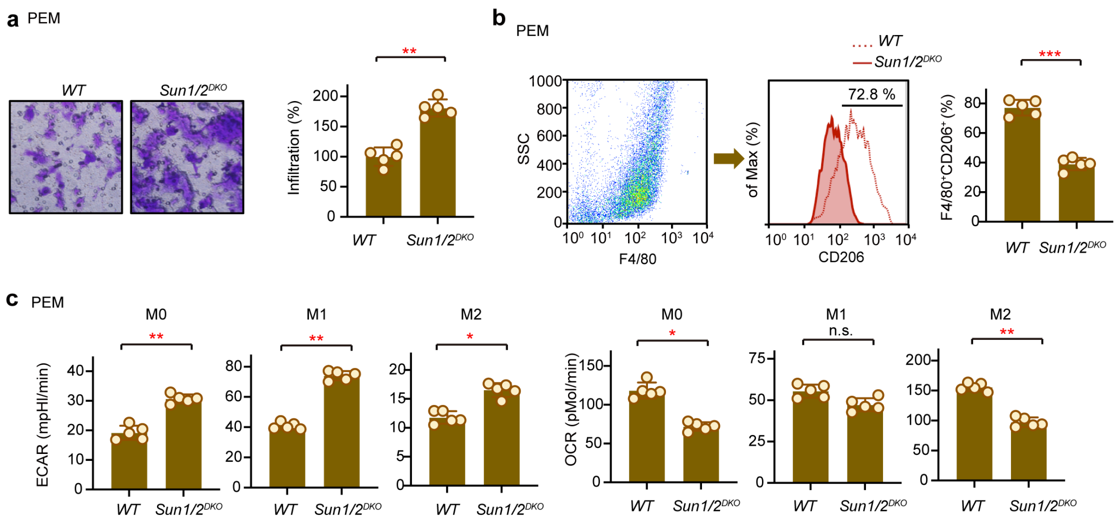

Supplementary Fig. 6 Depletion of SUN1/2 promotes M1 but suppresses M2 polarization of macrophages. a Cell infiltration of *WT* and *Sun1/2*^{DKO} PEMs. Infiltrated cells were stained with 1% crystal violet and photographed under a light microscope (n=5 biological replications per group). For quantification, infiltrated cells were soaked in 200 µl of DMSO and subjected to optical density measurement at 450 nm using DMSO as a blank control. OD450 from wildtype cells was calculated as 100% and then relative percentage of SUN1/2 knockout cells were calculated. **b** Flow cytometry analysis of the proportions of M2 macrophages (F4/80^{+}CD206^{+}) from *WT* and *Sun1/2*^{DKO} mice (n=5 mice per group). **c** Basal ECAR and OCR of M0, M1 and M2 macrophages, measured in real time and presented as mpH or pmol per unit time (n=5 biological replications per group). Wildtype, WT. Data were presented as means±SD (a-c). Two-sided unpaired student's *t*-test was used to compare difference between two groups (a-c). *, p<0.05; **, p<0.01; ***, p<0.001, *n.s.*, no significance in comparison with control group. Related to Fig. 6

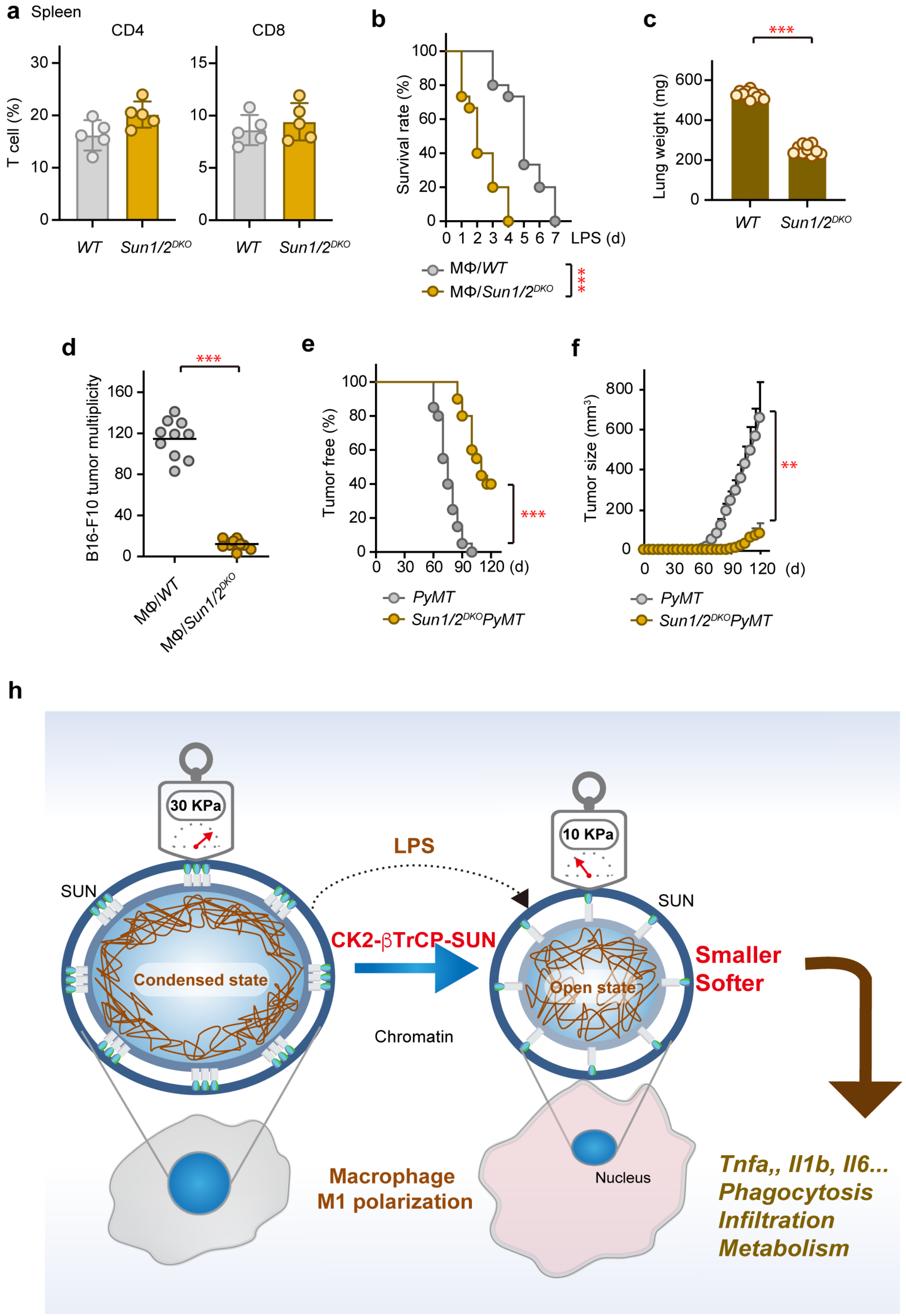

Supplementary Fig. 7 Depletion of SUN1/2 in macrophages enhances inflammation and antitumor immunity in mice. a Bar graph showing the proportion of $CD4^+$ and $CD8^+$ populations in wildtype and *Sun1/2*^{DKO} mice (n=4 per group). **b** Survival rate of LPS-treated mice after they received macrophages from *WT* and *Sun1/2*^{DKO} mice (n=10 mice per group). **c**

Weight of lungs from *WT* and *Sun1/2*^{DKO} mice after injecting with B16-F10 cells (n=10 mice per group). **d** Tumor burden of B16-F10-treated mice after they received macrophages from *WT* and *Sun1/2*^{DKO} mice (n=10 mice per group). The horizonal line corresponds to the mean value. **e** Tumor-free survival of *PyMT* and *Sun1/2*^{DKO}*PyMT* mice. **f** Tumor size in *PyMT* and *Sun1/2*^{DKO}*PyMT* mice (n=10 mice per group). The tumor size was measured every five days. **h** Nuclear size and stiffness are both acutely reduced during M1 polarization of macrophages. Macrophage, MΦ. Data are presented as mean ± SD (a,c). Data are presented as mean + SD (f). Two-sided unpaired student's *t*-test was used to compare difference between two groups (a,c,d,f). Kaplan Meier curve was performed using the logrank test (b,e). **, p<0.01; ***, p<0.001 in comparison with control group. Related to Fig. 7
