# Supplementary material for: SUN1/2 controls macrophage polarization via modulating nuclear size and stiffness
Source: Nat Commun. 2023 Oct 12;14:6416. doi: 10.1038/s41467-023-42187-5 (PMC10570371; doi:10.1038/s41467-023-42187-5)
Supplement: Supplementary file 2 — Description of Additional Supplementary Files [file 41467_2023_42187_MOESM2_ESM.pdf]

Description of Additional Supplementary Files

Supplementary Movie 1: The nucleus of PEM treated with PBS as a control.

Supplementary Movie 2: The nucleus of PEM treated with LPS.
