# Supplementary material for: SUN1/2 controls macrophage polarization via modulating nuclear size and stiffness
Source: Nat Commun. 2023 Oct 12;14:6416. doi: 10.1038/s41467-023-42187-5 (PMC10570371; doi:10.1038/s41467-023-42187-5)
Supplement: Supplementary file 5 — Reporting Summary [file 41467_2023_42187_MOESM5_ESM.pdf]

nature portfolio

Corresponding author(s): Zhaocai Zhou

Last updated by author(s): Sep 12, 2023

Reporting Summary

Nature Portfolio wishes to improve the reproducibility of the work that we publish. This form provides structure for consistency and transparency in reporting. For further information on Nature Portfolio policies, see our Editorial Policies and the Editorial Policy Checklist.

Statistics

For all statistical analyses, confirm that the following items are present in the figure legend, table legend, main text, or Methods section.

n/a	Confirmed	
☐	☒	The exact sample size (*n*) for each experimental group/condition, given as a discrete number and unit of measurement
☐	☒	A statement on whether measurements were taken from distinct samples or whether the same sample was measured repeatedly
☐	☒	The statistical test(s) used AND whether they are one- or two-sided<br>*Only common tests should be described solely by name; describe more complex techniques in the Methods section.*
☐	☒	A description of all covariates tested
☐	☒	A description of any assumptions or corrections, such as tests of normality and adjustment for multiple comparisons
☐	☒	A full description of the statistical parameters including central tendency (e.g. means) or other basic estimates (e.g. regression coefficient) AND variation (e.g. standard deviation) or associated estimates of uncertainty (e.g. confidence intervals)
☐	☒	For null hypothesis testing, the test statistic (e.g. *F*, *t*, *r*) with confidence intervals, effect sizes, degrees of freedom and *P* value noted<br>*Give P values as exact values whenever suitable.*
☒	☐	For Bayesian analysis, information on the choice of priors and Markov chain Monte Carlo settings
☒	☐	For hierarchical and complex designs, identification of the appropriate level for tests and full reporting of outcomes
☒	☐	Estimates of effect sizes (e.g. Cohen's *d*, Pearson's *r*), indicating how they were calculated

Our web collection on statistics for biologists contains articles on many of the points above.

Software and code

Policy information about availability of computer code

Data collection	No software was used for data collection.
Data analysis	n/a

For manuscripts utilizing custom algorithms or software that are central to the research but not yet described in published literature, software must be made available to editors and reviewers. We strongly encourage code deposition in a community repository (e.g. GitHub). See the Nature Portfolio guidelines for submitting code & software for further information.

Data

Policy information about availability of data

All manuscripts must include a data availability statement. This statement should provide the following information, where applicable:

- Accession codes, unique identifiers, or web links for publicly available datasets
- A description of any restrictions on data availability
- For clinical datasets or third party data, please ensure that the statement adheres to our policy

The sequencing data generated in this study have been deposited in the Gene Expression Omnibus (GEO) under accession code GSE85022.

Research involving human participants, their data, or biological material

Policy information about studies with human participants or human data. See also policy information about sex, gender (identity/presentation), and sexual orientation and race, ethnicity and racism.

Reporting on sex and gender	n/a
Reporting on race, ethnicity, or other socially relevant groupings	n/a
Population characteristics	n/a
Recruitment	n/a
Ethics oversight	n/a

Note that full information on the approval of the study protocol must also be provided in the manuscript.

Field-specific reporting

Please select the one below that is the best fit for your research. If you are not sure, read the appropriate sections before making your selection.

[x] Life sciences [ ] Behavioural & social sciences [ ] Ecological, evolutionary & environmental sciences

For a reference copy of the document with all sections, see nature.com/documents/nr-reporting-summary-flat.pdf

Life sciences study design

All studies must disclose on these points even when the disclosure is negative.

Sample size	No statistical methods were used to predetermine sample sizes and sample sizes were chosen empirically.
Data exclusions	No data were excluded from the analyses.
Replication	At least two independent experiments were performed with similar results.
Randomization	For in vivo experiments, all mice were randomly allocated into different experimental groups. For in vitro studies, no randomization was performed.
Blinding	The investigators were not blinded to allocation during experiments and outcome assessment.

Reporting for specific materials, systems and methods

We require information from authors about some types of materials, experimental systems and methods used in many studies. Here, indicate whether each material, system or method listed is relevant to your study. If you are not sure if a list item applies to your research, read the appropriate section before selecting a response.

Materials & experimental systems

n/a	Involved in the study	
[ ]	[x]	Antibodies
[ ]	[x]	Eukaryotic cell lines
[x]	[ ]	Palaeontology and archaeology
[ ]	[x]	Animals and other organisms
[x]	[ ]	Clinical data
[x]	[ ]	Dual use research of concern
[x]	[ ]	Plants

Methods

n/a	Involved in the study	
[x]	[ ]	ChIP-seq
[ ]	[x]	Flow cytometry
[x]	[ ]	MRI-based neuroimaging

Antibodies

Antibodies used	The primary antibodies and dilutions used were: Flag (Sigma, F3165, 1:2000), α-tubulin (Sigma, T6199, 1:2000) and β-actin (Sigma, A2228, 1:5000), β-TrCP(Cell Signaling Technology, 4394, 1:1000), Nesprin1 (Santa Cruz, sc-99065, 1:500), Nesprin2 (Santa Cruz, sc-365097, 1:500), LaminA/C (Santa Cruz, sc-7292, 1:1000), ubiquitin (Santa Cruz, sc-8017, 1:1000), human SUN1 (Abcam, ab103021, 1:1000), mouse SUN1 (Abcam, ab124770, 1:000) and mouse SUN2 (Abcam, ab124916, 1:1000), mouse SUN2 (Abcam, ab198981, 1:100). An antibody specific for human SUN2 (1:1000) was produced by Shanghai Immune Biotech (Shanghai, China).

Validation	Flag (Sigma, F3165, used in Western Blot, immunoprecipitation, immunofluorescence), α-tubulin (Sigma, T6199, immunocytofluorescence, Western Blot), β-actin (Sigma, A2228, Western Blot), β-TrCP (Cell Signaling Technology, 4394, Western Blot), Nesprin1 (Santa Cruz, sc-99065, Western Blot), Nesprin2 (Santa Cruz, sc-365097, Western Blot), LaminA/C (Santa Cruz, sc-7292, Western Blot), ubiquitin (Santa Cruz, sc-8017, Western Blot), human SUN1 (Abcam, ab103021, Western Blot, immunoprecipitation), mouse SUN1 (Abcam, ab124770, Western Blot, immunoprecipitation, immunofluorescence) and mouse SUN2 (Abcam, ab124916, Western Blot, immunoprecipitation, immunofluorescence).

Eukaryotic cell lines

Policy information about cell lines and Sex and Gender in Research

Cell line source(s)	THP-1 cells and HEK293T cells were purchased from the Cell Resource Center of the Institute of Life Sciences, Chinese Academy of Sciences (Shanghai, China). Human bone marrow-derived macrophages (hBMs, CP-H186) were purchased from Procell (Wuhan, China). B16-F10 murine melanoma cells were kindly provided by Prof. Xiaolong Liu. PEMs were isolated from mice by applying a peritoneal lavage four days after i.p. injection of 1 ml of 3% thioglycollate. Cells (2×106) were then obtained from the peritoneal cavities of mice, plated for 2 h in 33 mm cell culture dishes, which were then extensively washed to eliminate cells that did not attach. Bone marrow-derived macrophages (BMDMs) were obtained by flushing tibiae and femurs from mice with ice-cold PBS and passing the suspension through a cell strainer with a 70m cut-off. Cells (2×106) were plated on 33 mm cell culture dishes in 10 ml complete cultures supplemented with granulocyte-macrophage colony-stimulating factor (GM-CSF) or macrophage colony-stimulating factor (M-CSF) for seven days. THP-1 cells, B16-F10, PEMs and BMDMs were maintained in RPMI1640 medium, whereas HEK293T and MEF cells were in DMEM medium.
Authentication	All cell lines were not authenticated.
Mycoplasma contamination	All cell cultures supplemented with 10% FBS, L-glutamine and penicillin/streptomycin were routinely checked for mycoplasma contamination by using MycoAlert™ Mycoplasma Detection Kit (LT07-318, Lonza, Rockland, ME).
Commonly misidentified lines (See ICLAC register)	No commonly misidentified cell lines were used in the study.

Animals and other research organisms

Policy information about studies involving animals; ARRIVE guidelines recommended for reporting animal research, and Sex and Gender in Research

Laboratory animals	Sun1fl/flSun2-/- mice (C57BL/6 background) were kindly provided from Prof. Min Han. MMTV-PyMT transgenic mice and LysMcre/+ mice were obtained from the Jackson laboratory. To generate mice with macrophage SUN1/2 deficiency, we crossed SUN1fl/flSUN2-/- mice with LysMcre/wt mice to obtain Sun1fl/flSun2-/-LysMcre/cre mice (Sun1/2DKO mice) and their Sun1fl/flSun2+/+ littermates (WT mice).
Wild animals	Mice (C57BL/6 background) used in this study were from SLAC Laboratory Animal (Shanghai, China)
Reporting on sex	For all experiments adult animals (not selected for gender) with a minimum age of 6 weeks were used.
Field-collected samples	The mice were housed under specific pathogen-free conditions in automated watered and ventilated cages on a 12-h light/dark cycle.
Ethics oversight	Animal procedures were approved by the Institutional Animal Care and Use Committee of Fudan University (approval ID, IDM2022037) and the Institutional Animal Care and Use Committee of the Institute of Center for Excellence in Molecular Cell Science (approval ID: SIBCB-NAF-14-004-S329-023).

Note that full information on the approval of the study protocol must also be provided in the manuscript.

Flow Cytometry

Plots

Confirm that:

- [x] The axis labels state the marker and fluorochrome used (e.g. CD4-FITC).
- [ ] The axis scales are clearly visible. Include numbers along axes only for bottom left plot of group (a 'group' is an analysis of identical markers).
- [ ] All plots are contour plots with outliers or pseudocolor plots.
- [ ] A numerical value for number of cells or percentage (with statistics) is provided.

Methodology

Sample preparation	Cells were put into a 5 ml polystyrene round-bottom tube and stained for 30 min at 4ºC with indicated antibodies. Intracellular staining was performed after 10 min fixation (2% formaldehyde PBS) at room temperature and 5 min permeabilization in IC staining buffer (0.1% saponin, 0.1% bovine serum albumin Hank's balanced salt solution) at 4ºC.

Instrument	Cell fluorescence was determined using a two-laser FACS Calibur (BD Biosciences, Mississauga, ON, Canada) flow cytometer.
Software	Data were analyzed with FlowJo software (TreeStar, Olten, Switzerland).
Cell population abundance	Cell purity was consistently greater than 90%
Gating strategy	Gating strategy for macrophages was shown in Supplementary Fig. 2b.

☒ Tick this box to confirm that a figure exemplifying the gating strategy is provided in the Supplementary Information.
